# Synthesis of 1,3,5-Triazepines and Benzo[*f*][1,3,5]triazepines and Their Biological Activity: Recent Advances and New Approaches

**DOI:** 10.3390/molecules29030632

**Published:** 2024-01-29

**Authors:** Ameen Ali Abu-Hashem, Othman Hakami, Nasser Amri, Yousef E. Mukhrish, Ahmed A. M. Abdelgawad

**Affiliations:** Department of Physical Sciences, Chemistry Division, College of Science, Jazan University, P.O. Box 114, Jazan 45142, Saudi Arabia; omhakami@jazanu.edu.sa (O.H.); yemukhrish@jazanu.edu.sa (Y.E.M.); ahmedawad26@hotmail.com (A.A.M.A.)

**Keywords:** benzo[*f*][1,3,5]triazepine, *o*-phenylenediamine, 2-aminobenzamide, pyrazoles, isothiocyanates, thiazoles, oxadiazoles, oxadiazepines, hydrazonoyl chloride, cyclization, alkylation, condensation, antiviral, psychotropic, anticancer activity

## Abstract

This review article discusses the recent progress in synthesizing seven-membered ring 1,3,5-triazepine and benzo[*f*][1,3,5]triazepine derivatives. These derivatives can be either unsaturated, saturated, fused, or separated. This review covers strategies and procedures developed over the past two decades, including cyclo-condensation, cyclization, methylation, chlorination, alkylation, addition, cross-coupling, ring expansions, and ring-closing metathesis. This review discusses the synthesis of 1,3,5-triazepine derivatives using nucleophilic or electrophilic substitution reactions with various reagents such as *o*-phenylenediamine, 2-aminobenzamide, isothiocyanates, pyrazoles, thiazoles, oxadiazoles, oxadiazepines, and hydrazonoyl chloride. This article systematically presents new approaches and techniques for preparing these compounds. It also highlights the biological importance of benzo[*f*][1,3,5]triazepine derivatives, which have been used as drugs for treating nervous system diseases. This review aims to provide researchers with the necessary information to create and develop new derivatives of these compounds as quickly as possible.

## 1. Introduction

Seven-membered heterocyclic rings are molecules that contain nitrogen atoms and exhibit significant biological activities [1,2,3]. They are commonly found in natural products and bioactive pharmaceuticals. Therefore, many attempts have been made to synthesize them [4,5,6]. All compounds with triazepine structures have gained attention for their unique biological properties [7,8]. Triazepines are interesting both pharmacologically and chemically [9,10,11,12]. Triazepine derivatives are a type of seven-membered heterocyclic compound with various pharmacological activities, such as antibacterial, antiviral, psychotropic, anticancer, CCK2 antagonist, antisecretory, anti-inflammatory, and analgesic activities [13,14,15,16,17,18,19,20]. Moreover, the combination of a pyrimidine and triazepine moiety exhibits enhanced pharmacological effects such as antiviral, antifungal [21], and antidiabetic [22], and functions as an inhibitor [23].

**Figure 1:**
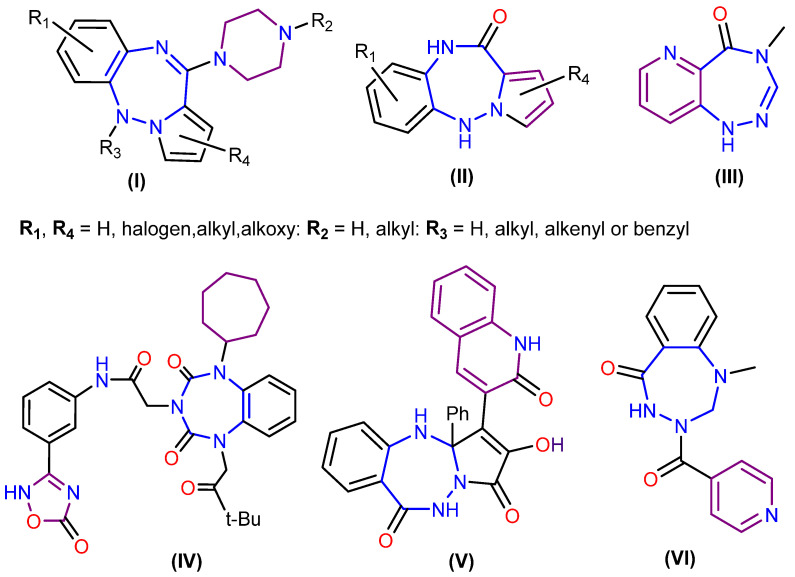
Derivatives of triazepine that are biologically active.

The benzotriazepine class is of great interest due to its ability to affect the central nervous system and its use as psychoactive agents. For example, compounds (**I**) and (**II**) are effective neuroleptic agents used to treat psychotic disorders such as schizophrenia [14]. Compound (**III**) exhibits moderate antisecretory activity in rats [15], while compound (**IV**) acts as a CCK2 receptor antagonist with better selectivity than CCK1 receptors [17]. Compound (**V**) has been found to have analgesic activity in white mongrel mice [19]. Finally, compound (**VI**) has shown antipsychotic activity comparable to that of the reference drug clozapine [20], as demonstrated in (Figure 1).

It has recently been discovered that seven-membered ring heterocycles have significant biological activities [24,25,26], especially considering the increasing cancer problem worldwide. Chemotherapy is often not completely effective due to its toxicity to other tissues. As a result, scientific researchers have turned their attention to developing various pharmaceutical drugs. Benzodiazepine derivatives are widely used in many pharmaceutical drugs, including alprazolam, bromazepam, clorazepate, and diazepam (valium).

**Figure 2:**
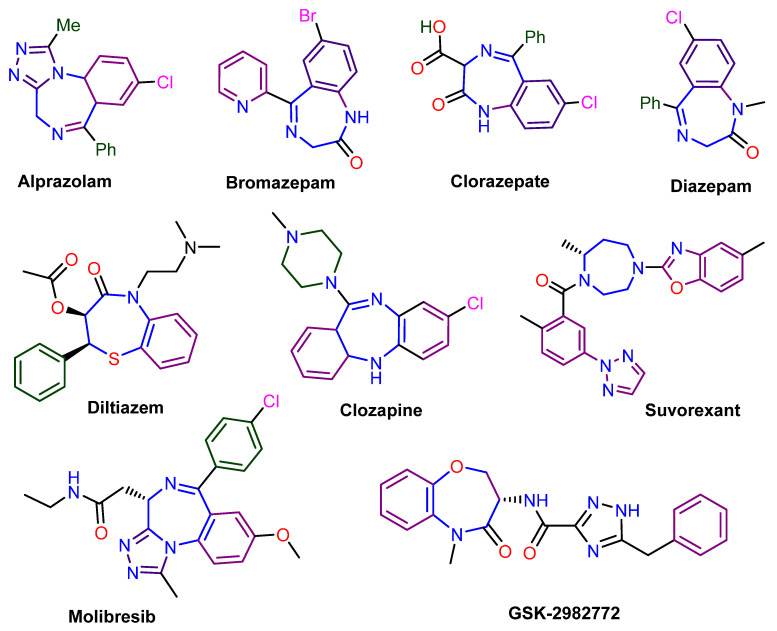
The benzodiazepine drug derivatives.

Benzodiazepine derivatives such as oxazolam, prazepam, and clorazepate are used to treat various conditions like panic disorders, anxiety, muscle spasms, restless legs syndrome, seizures, and trouble sleeping. These drugs have multiple biological activities that include anti-HIV agents [27], antiulcers [28], and antibiotics [29]. They are also anxiolytics, anticonvulsants, sedatives, skeletal muscle relaxants, and hypnotics [30]. Recent studies have shown that the diazepine moiety present in these drugs has several biological activities such as the inhibition of platelet aggregation [30], as well as being protein kinase inhibitors [31], H3 receptor antagonists [32], 5-HT antagonists [33,34], peptidomimetics [35], anti-HIV agents [36], and matrix metalloproteinase inhibitors [37]. Also, these drugs possess DNA strand-breaking activity [38] and their use demonstrates the importance of seven-membered rings in drug design [39], as shown in (Figure 2).

**Figure 3:**
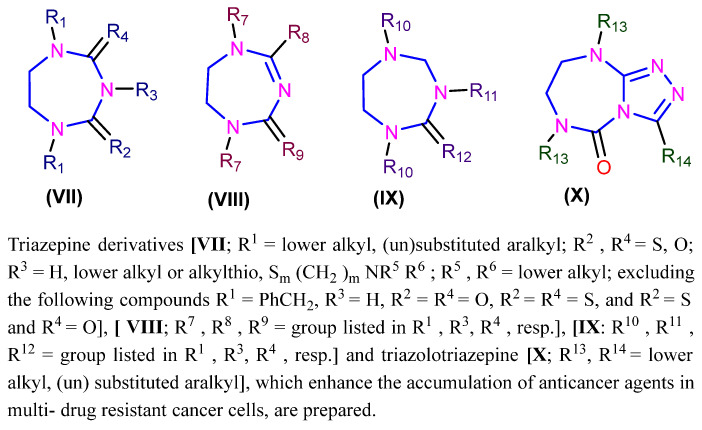
The 1,3,5-triazepine derivatives that act as anticancer agents.

Cancer has become a widespread disease among humans in recent years. As a result, many scientists have been working hard to find a cure for this disease. They have been preparing numerous heterocyclic compounds and studying their effects and toxicity on cancer cells. Among these compounds are the 1,3,5-triazepine and 1,2,4-triazolo[3,4-b][1,3,5]triazepine derivatives that have been found to be effective as anticancer agents [40]. You can find additional details in (Figure 3), which displays the reagents as follows: 0.015 mol *N*,*N*′-bis(p-chlorobenzyl) ethylenediamine and 0.015 mol Et carboethoxy carbamate were refluxed in xylene for 5 h to give a triazepine-dione derivative **VII** (R^1^ = p-chlorobenzyl, R^2^ = R^4^ = O, and R^3^ = H) which was refluxed with Lawesson’s reagent in toluene to give triazepinethione **VIII** (R^1^ = p-chlorobenzyl, R^2^ = S, R^3^ = H, and R^4^ = O) (V). [^3^H]-vinblastine at 10 n M showed a 14.0-fold increase in the accumulation of adriamycin-resistant P388/ADR cells in the presence of 50 μ M V. Pharmaceutical formulations, e.g., tablets containing V, were prepared as detailed in [40] (Figure 3).

In this review article, different safe and environmentally friendly methods for preparing 1,3,5-triazepine derivatives have been discussed. These methods are cost-effective, time-saving, and have potential applications in both medical and industrial fields. By adopting these methods, we can achieve sustainable development in all aspects of society. Thus, different methods for synthesizing fused triazepines are described in the literature [41,42].

As part of our research efforts, we have published several scientific articles to benefit the environment and society. These articles focus primarily on synthesizing new heterocyclic compounds and investigating their biological and pharmacological properties [43,44,45,46,47,48,49,50,51,52,53]. In this review article, we delve into the synthesis methods, chemical reactions, and physical and pharmacological activity of 1,3,5-triazepine and benzo[*f*][1,3,5]triazepine derivatives with a specific focus on seven-membered heterocyclic rings.

## 2. Synthesis of 1,3,5-Triazepines and Benzo[*f*][1,3,5]triazepines with Biological Activity

### 2.1. From o-Phenylenediamine

**Scheme 1:**
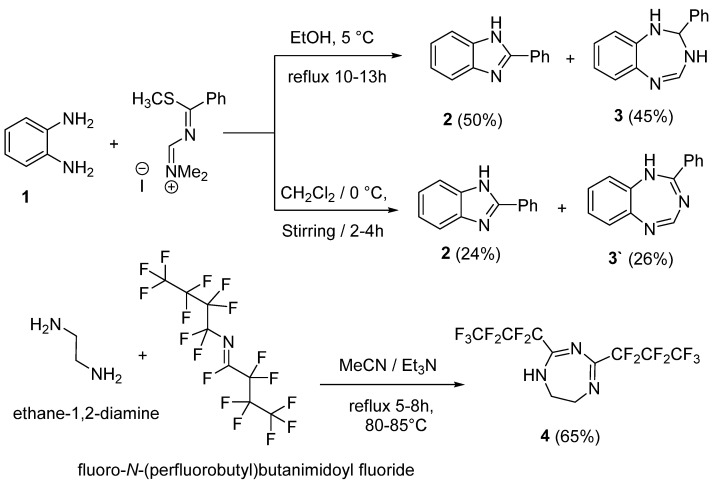
Synthesis of benzo[*d*]imidazole and 2-phenyl-benzo[*f*][1,3,5]triazepine derivatives.

Substituted aromatic ketones (acetophenone) and aldehydes condensate to form chalcones. These react with *o*-phenylenediamine (**1**) to yield benzodiazepine derivatives which can be converted to benzimidazole and benzotriazepine derivatives under basic conditions. Therefore, condensing *o*-phenylenediamine (**1**) and *N*-methyl-*N*-(methylthio) (phenyl) methylene) amino) methylene) methanaminium iodide as amidinium salt in ethanol at 5 °C or dichloromethane at 0 °C yielded 2-phenyl-1*H*-benzo[*d*]imidazole (**2**) and 2-phenyl-2,3-dihydro-1*H*-benzo[*f*][1,3,5]triazepine (**3**) or 2-phenyl-1*H*-benzo[*f*][1,3,5]triazepine (**3′**).The compound 2-phenyl-1,3-benzimidazole (**2**) was produced when *o*-phenylenediamine (**1**) attacked the C_3_ of amidinium salt. On the other hand, 2-phenyl-1,3,5-benzotriazepine (**3**) was formed when *o*-phenylenediamine attacked the C_3_ and C_1_ of amidinium salt, resulting in a double attack. However, at this temperature, the major product obtained was 2-phenyl-1,3-benzimidazole (**2**), which was isolated in moderate yield (60%). Also, the reaction between ethane-1,2-diamine and 2,2,3,3,4,4,4-heptafluoro-*N*-(perfluorobutyl) butanimidoyl fluoride in acetonitrile, in the presence of triethylamine, resulted in the formation of 2,4-bis(perfluoropropyl)-6,7-dihydro-1*H*-1,3,5-triazepine (**4**), which is a seven-membered heterocycle, and these compounds have diverse biological activities [54,55,56], as shown in Scheme 1.

**Scheme 2:**
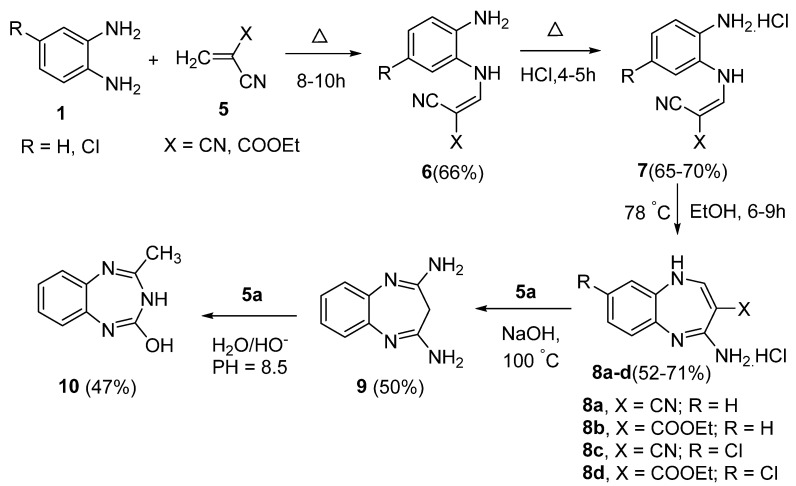
Synthesis of benzo[*b*][1,4]diazepine and benzo[*f*][1,3,5]triazepine derivative.

The compound benzene-1,2-diamine (**1**) reacted with 2-methylene-malononitrile (**5**) to produce 2-((2-amino phenyl) amino) methylene) malononitrile (**6**). Further, refluxing the compound (**6**) in the presence of hydrochloric acid resulted in the formation of 2-(((2-aminophenyl) amino) methylene) malononitrile hydrochloride (**7**). Upon refluxing compound (**7**) in absolute ethanol, it cyclized to yield 4-amino-1H-benzo[*b*][1,4]diazepine-3-carbonitrile hydrochloride derivatives (**8a**–**d**). Compound (**8a**) was hydrolyzed with bases in a solution of sodium hydroxide to form diazepine, 3*H*-benzo[*b*][1,4]diazepine-2,4-diamine (**9**), which was further converted into 4-methyl-benzo[*f*][1,3,5]triazepin-2-ol (**10**) [57], as shown in Scheme 2.

**Scheme 3:**
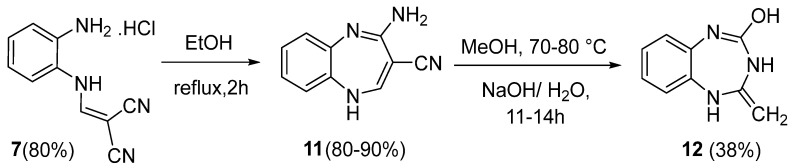
Synthesis of 4-amino-benzo[*b*][1,4]diazepine-3-carbonitrile/2-methylene-benzo[*f*][1,3,5]triazepin-4-oles.

When hydrochloric acid is present, *o*-*N*-(dicyano-vinyl) amino-aniline can be easily converted into 4-amino-1,5-benzodiazepine-3-carbonitrile. This compound can be hydrolyzed with various bases to produce other derivatives such as diazepine, triazepine, or benzimidazole. Refluxing the *o*-*N*-(dicyano-vinyl) amino-aniline hydrochloride (**7**) in ethanol results in precipitation from H_2_O (orange needles, yield of 80–90%,) of 4-amino-1H-benzo[*b*][1,4]diazepine-3-carbonitrile (**11**). The 2-methylene-2,3-dihydro-1*H*-benzo[*f*][1,3,5]triazepin-4-ol (**12**) was obtained by hydrolyzing compound (**11**) with sodium hydroxide in water [58]. It should be noted that the compound formed at a temperature range of 70–80 °C, as shown in Scheme 3.

**Scheme 4:**
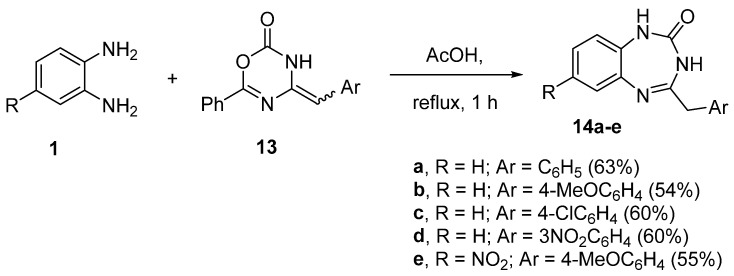
Synthesis of 4-(substituted-benzyl)-benzo[*f*][1,3,5]triazepin-2-one’s derivatives.

The synthesis of 4-(substituted-benzyl)-1,3-dihydro-2*H*-benzo[*f*][1,3,5]triazepin-2-ones (**14**) from *o*-phenylenediamines (**1**) and 4-(arylmethylene)-6-phenyl-3,4-dihydro-2*H*-1,3,5-oxadiazin-2-one (**13**) [59] is shown in Scheme 4.

**Scheme 5:**
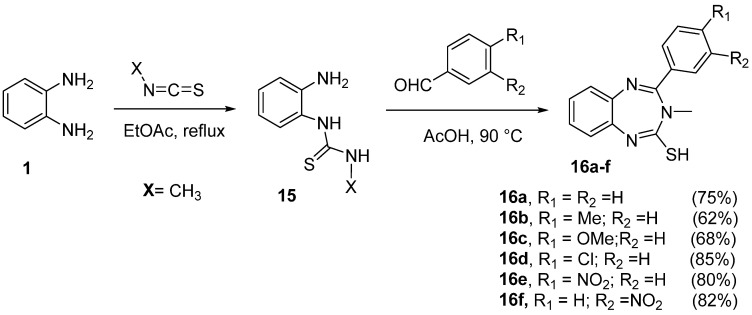
Synthesis of substituted-phenyl-3*H*-benzo[*f*][1,3,5]triazepine-2-thiol derivatives.

In the reaction between *o*-phenylenediamines (**1**) and methyl-isothiocyanate, a process of cyclo-condensation, dehydrogenation, and cyclization occurs, resulting in the formation of 1-(2-aminophenyl)-3-methylthiourea (**15**). This compound (**15**) has been reacted with aromatic aldehydes in acetic acid to yield 3-methyl-4-substituted-phenyl-3*H*-benzo[*f*][1,3,5]triazepine-2-thiol (**16**) derivatives [60], as shown in Scheme 5.

**Scheme 6:**
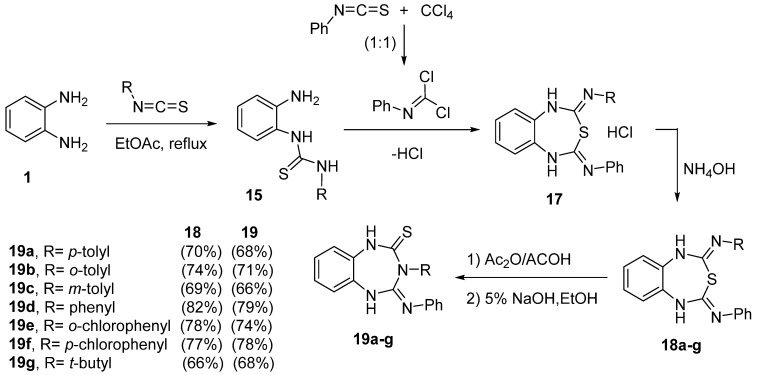
Synthesis of 1,5-dihydrobenzo[*d*][1,3,6]thiadiazepine-2,4-diimine/3-substituted-4-(phenylimino)-1,3,4,5-tetrahydro-2H-benzo[*f*][1,3,5]triazepine-2-thione derivatives.

Benzo-[4,5-*d*]-2-phenylimino-7-aryl/alkylimino-1,3,6-thiadiazepines (**18**) were synthesized by basifying benzo-(4,5-*d*)-2-phenylimino-7-arylialkylimino-1,3,6-thiadiazepine hydrochlorides (**17**). The hydrochlorides (**17**) were prepared by reacting *N*-phenyl isocyanate dichloride with 1-arylalkyl-3-(2′-amino) phenyl thiocarbamides (**15**), which were initially made by condensing aryl/alkyl isothiocyanates and *o*-phenylenediamine. Acylating of *N*^2^,*N*^4^-substituted-phenyl-1,5-dihydrobenzo[*d*][1,3,6]thiadiazepine-2,4-diimine (**18**) with acetic anhydride and glacial acetic acid at a 1:2 ratio resulted in 3,6-diacetyl intermediate derivatives. Boiling these intermediate derivatives with an aqueous ethanolic solution of sodium hydroxide isomerized them into the corresponding 3-substituted-4-(phenylimino)-1,3,4,5-tetrahydro-2*H*-benzo[*f*][1,3,5]triazepine-2-thione (**19**) derivatives. Gram-positive and Gram-negative microorganisms including *S. aureus*, *E. coli*, *S. typhi*, *A. aerogenes*, *B. subtilis*, and *A. niger* were tested for antimicrobial activity using these compounds (**18a**–**g**) and (**19a**–**g**) [61], as shown in Scheme 6.

**Scheme 7:**
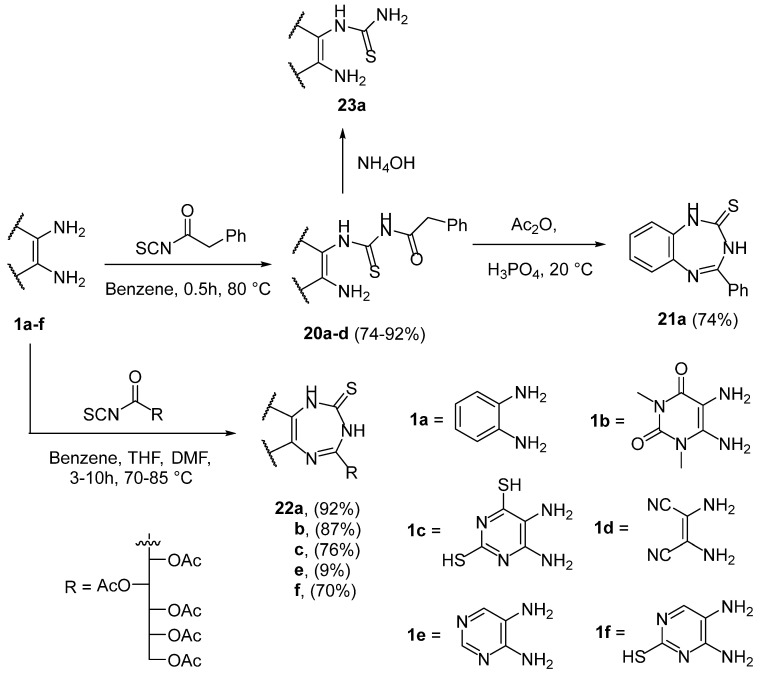
Synthesis of 4-phenyl-1,3-dihydro-2*H*-benzo[*f*][1,3,5]triazepine-2-thione/D-gluco-pentyl benzotriazepine-2-thione/D-gluco-pentyl-pyrimidotriazepine-2-thiones derivatives.

The chemical reaction between phenylacetyl isothiocyanate and diamines produces 1-substituted 3-phenylacetyl thiourea (**20a**–**d**) in high yields. However, trying to close the ring of these products with thermal or basic conditions failed. On the other hand, treating (**20a**) with Ac_2_O-H_3_PO_4_ at room temperature led to the formation of the cyclized product 4-phenyl-1,3-dihydro-2*H*-benzo[*f*][1,3,5]triazepine-2-thione (**21a**). Similarly, reacting D-gluconyl isothiocyanate with *o*-phenylenediamine (**1a**) or diaminopyrimidines (**1b**, **c**, **e**, **f**) produced D-gluco-pentyl benzo-triazepine-2-thione (**22a**) or D-gluco-pentyl-pyrimidotriazepine-2-thiones (**22b**, **c**, **e**, **f**), respectively, in moderate yields, Also, the reaction of (**20b**) with ammonium hydroxide gave 1-(6-amino-1,3-dimethyl-2,4-dioxopyrimidin-5-yl)thiourea (**23a**) in a quantitative yield [62], as depicted in Scheme 7.

### 2.2. From 2-(Imidazolidin-2-ylideneamino) Aniline Derivatives

**Scheme 8:**
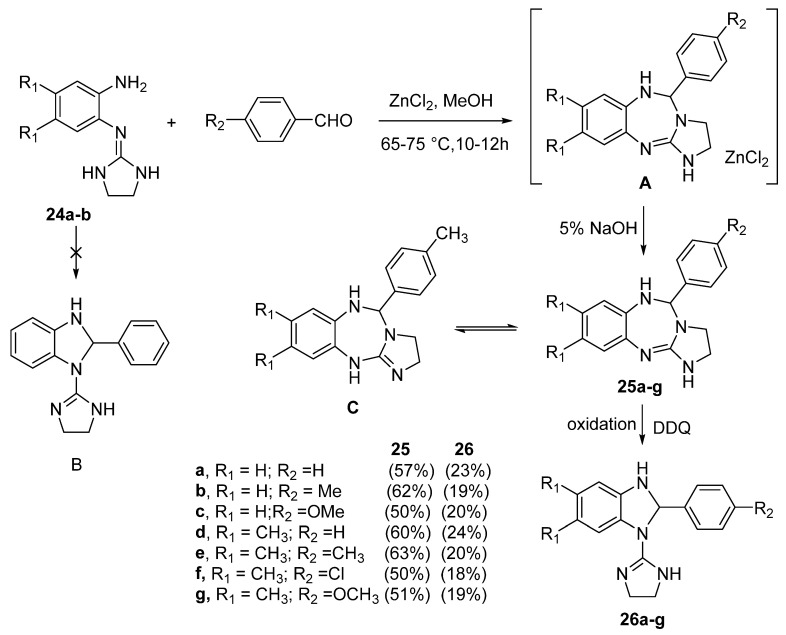
Synthesis of 5-phenyl-1*H*-benzo[*f*]imidazo[2,1-*b*][1,3,5]triazepine/1-(imidazol-2-yl)-2-phenyl-benzo[*d*]imidazole by oxidation of triazepine ring contraction derivatives.

The chemical synthesis of 5-aryl-2,3,5,6-tetrahydro-3*H*-imidazo[2,1-*b*][1,3,5]benzotriazepine derivatives (**25a**–**g**) was carried out by reacting 2-(2-aminoarylimino) imidazolidines (**24a**,**b**) with corresponding aryl aldehydes. When the compounds (**25**) containing the aminal group were treated with 2,3-dichloro-5,6-dicyano-1,2-benzoquinone (DDQ), they underwent oxidation ring contraction, leading to the formation of 1-(4,5-dihydro-1*H*-imidazol-2-yl)-2-aryl-benzimidazoles (**26a**–**g**) [63], as shown in Scheme 8.

**Scheme 9:**
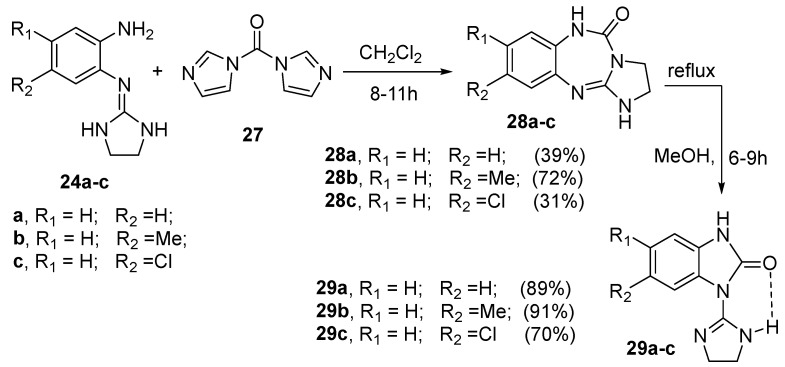
Synthesis of benzo[*f*]imidazo[2,1-*b*][1,3,5]triazepin-5-one/1-(imidazol-2-yl)-1,3-dihydro-2*H*-benzo[*d*]imidazol-2-ones.

The reaction of 2-(imidazolidin-2-ylideneamino) aniline derivatives (**24a**–**c**) with carbonyldiimidazole (**27**) (CDI) produced 2,3,5,6-tetrahydro-5*H*-benzo[*f*]imidazo[2,1-*b*][1,3,5] triazepin-5-one (**28a**–**c**). When heated in boiling methanol, it gave the corresponding 1-(4,5-dihydro-1*H*-imidazol-2-yl)-1,3-dihydro-2*H*-benzo[*d*]imidazol-2-one (**29a**–**c**) [63], as demonstrated in Scheme 9.

### 2.3. From 2-Aminobenzamide

Condensation of 2-methyl-/2-ethyl- and 2-phenyl-/p-tolyl-4-arylidene-/heterylmethylidene-2-oxazolin-5-ones (c-azlactones) (**31** and **31′**) with *o*-aminobenzamide (**30**) in acetic acid resulted in the formation of two entirely different heterocyclic systems of differently substituted quinazoline compounds by using intermediate (**32** and **32′**), 2-methyl-/2-ethyl-3-a-carboxy-a-styryl-/b-heteryl-a-carboxyvinyl-quinazolin-4(3*H*)-ones (**33a**–**e**) derivatives and differently substituted 1,4-benzodiazepine compounds, 3-arylidene-/heteryl methylidene-4-aroyl-1*H*-[1,4]benzodiazepine-2,5(3*H*,4*H*)-diones (**34a**–**e** and **35a**–**e**).

**Scheme 10:**
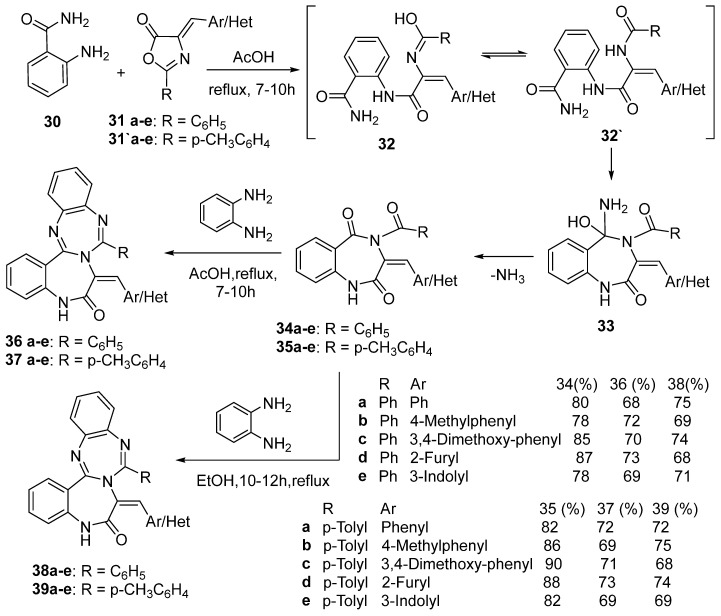
Synthesis of 7-substituted-benzylidene-9-phenyl-5*H*-benzo[*f*]benzo[5,6][1,4]diazepino [7,1-*b*][1,3,5]triazepinones.

Compounds (**34a**–**e** and **35a**–**e**) have been converted into compounds, (**36a**–**e** and **37a**–**e**; **38a**–**e**, and **39a**–**e**) through different transformations. Benzodiazepines, (**34a**–**e** and **35a**–**e**), through condensation with *o*-phenylenediamine, have generated heterocyclic systems (**36a**–**e** and **37a**–**e**; **38a**–**e** and **39a**–**e**) [64], as demonstrated in Scheme 10.

### 2.4. From Carbonyl and Di-Carbonyl Compounds

**Scheme 11:**
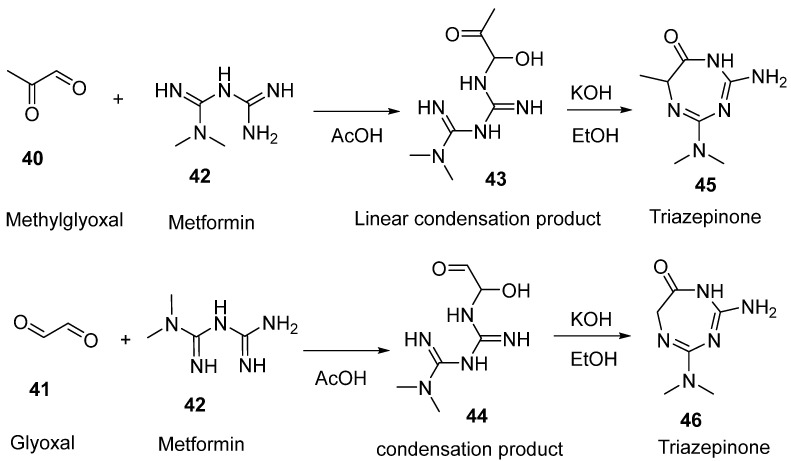
Synthesis of 4-amino-2-(dimethylamino)-7-methyl-1,3,5-triazepinone/4-amino-2-(dimethylamino)-1,3,5-triazepinone from metformin with methylglyoxal or glyoxal derivatives.

A derivative of metformin and methylglyoxal, called a metformin–methylglyoxal adduct (**43**), was created and identified as a triazepinone derivative. Two variations of the derivative were produced: 4-amino-2-(dimethylamino)-7-methyl-5,7-dihydro-6*H*-1,3,5-triazepin-6-one (**45**) and 4-amino-2-(dimethylamino)-5,7-dihydro-6*H*-1,3,5-triazepin-6-one (**46**). The results of the experiment showed that metformin (**42**) reacted with methylglyoxal (**40**) and glyoxal (**41**) to form guanidine–dicarbonyl adducts (**43** and **44**). Studies of the reaction kinetics, as well as the mass fragmentation spectra of the reaction products, indicated the presence of triazepinone derivatives (**45**, **46**). When metformin was present, the fluorescence of AGE-related compounds decreased by 37% and 45% after albumin incubation with glyoxal or methylglyoxal, respectively. Compounds (**42**–**46**) suggest that metformin could also reduce AGE formation by reacting with a di-carbonyl compound in addition to its antihyperglycemic effect. This finding is significant for the clinical application of metformin in preventing diabetic complications by inhibiting carbonyl stress [65,66,67]. This is demonstrated in Scheme 11.

**Scheme 12:**
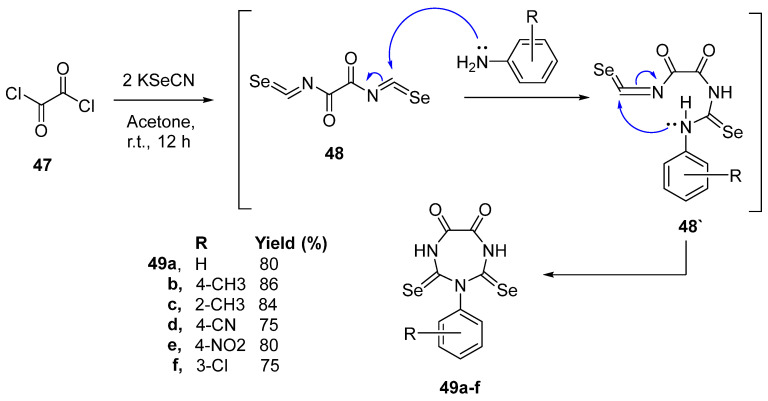
The reaction of substituted anilines, oxalyl chloride, and two molecules of potassium seleno-cyanate in acetone at room temperature give 3-substituted-phenyl-2,4-diselenoxo-1,3,5-triazepane-6,7-dione.

A series of 3-aryl-2,4-dithioxo-1,3,5-triazepane-6,7-diones can be synthesized by reacting oxalyl chloride, anilines, and two molecules of ammonium thiocyanate in acetone under ultrasound irradiation. We believe that similar analogues of 3-aryl-2,4-diselenoxo-1,3,5-triazepane-6,7-diones (**49**) can be synthesized by replacing ammonium thiocyanate with potassium selenocyanate. It was discovered that a series of compounds called 3-substituted-phenyl-2,4-diselenoxo-1,3,5-triazepane-6,7-dione (**49**) can be synthesized in good yield by reacting various substituted anilines (1; R = various) with oxalyl chloride (**47**) and KSeCN. This reaction gives rise to two intermediate compounds (**48** and **48′**) before the final product (**49**) is obtained [68]. There is a proposed tentative mechanism for a transformation. It is suggested that oxalyl diisoselenocyanate was created from the reaction of two equivalents of KSeCN with one equivalent of oxalyl chloride. This was followed by the addition of anilines to create (**48′**). Intermediate (**48′**) underwent subsequent cyclization, which was then converted into (**49**) by intramolecular cyclization (Scheme 12).

**Scheme 13:**
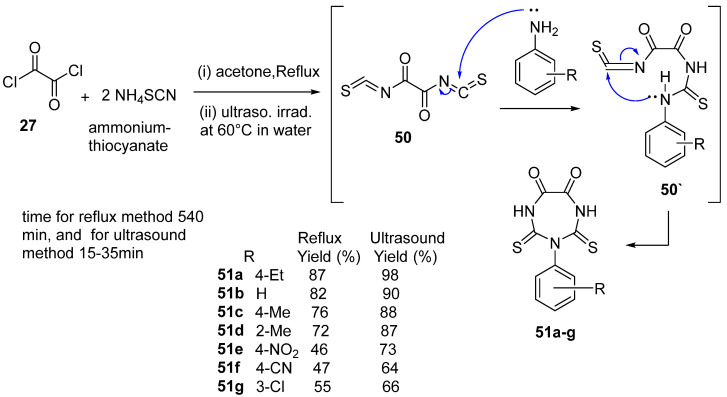
Synthesis of substituted-3-phenyl-2,4-dithioxo-1,3,5-triazepane-6,7-dione.

We have shown and described a four-component reaction using oxalyl chloride (**27**), aniline derivatives, and two molecules of ammonium thiocyanate in acetone under ultrasound irradiation to synthesize 3-aryl-2,4-dithioxo-1,3,5-triazepane-6,7-diones (**51**). Several triazepanes were synthesized by reacting oxalyl chloride (**27**) with different aniline derivatives and two equivalents of NH_4_SCN. The reaction was conducted under two different conditions: (i) reflux in acetone and (ii) ultrasound irradiation at 60 °C in water. The initial step of the reaction was carried out at 0–5 °C due to the fast release of chlorine atoms from oxalyl chloride. Oxalyl diisothiocyanate (**50**) was created by mixing two equivalents of NH_4_SCN with one equivalent of oxalyl chloride (**27**). The color of the mixture turned red during the reaction. Afterwards, (**50**) reacted with aniline derivatives to form intermediates (**50′**). These intermediates then underwent intramolecular cyclization, resulting in the production of 1,3,5-triazepane derivatives (**51a**–**g**). The reaction also changed color from red to brown [69]. Based on the MTT analysis and cellular images, the cyclic urea compounds (**51a**–**g**) showed higher toxicity compared to the control sample. This is because the selected cells used in this study are MKN-45 gastric adenocarcinoma cells. As a result, the synthesized compounds (**51a**–**g**) are more effective in inducing cancer cell toxicity than the Paclitaxel drug [69], as shown in Scheme 13.

**Scheme 14:**
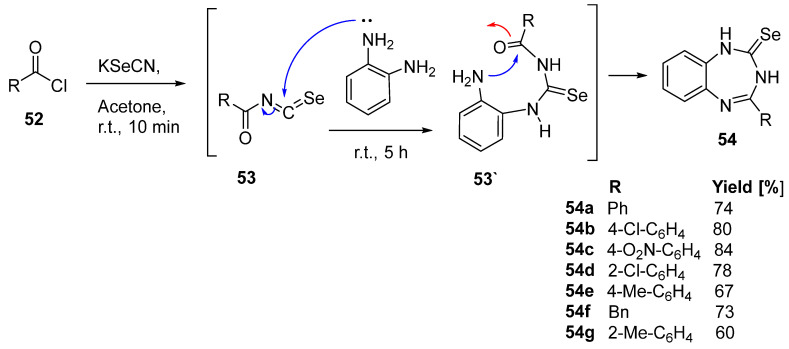
Synthesis of 4-Substituted 1,3-dihydro-2*H*-1,3,5-benzotriazepine-2-selone.

The reaction of acyl isoselenocyanates, produced from acyl chlorides and KSeCN, with benzene-1,2-diamine in acetone at room temperature produced 1,3,5-triazepineselone derivatives in moderate to good yields. A possible explanation for how 1,3,5-triazepineselone derivatives (**54**) are formed can be given as follows. It involves the acyl isoselenocyanate (**53**), which is created from (**52**) and KSeCN. This compound then reacts with *o*-phenylenediamine, resulting in the formation of an intermediate product (**53′**). Finally, this intermediate is transformed into the desired product, 4-phenyl-1,3-dihydro-2*H*-benzo[*f*][1,3,5]triazepine-2-selenone (**54**) derivatives, through cyclization and elimination of H_2_O [70]. In this study, benzene-1,2-diamine is used to react with acyl isoselenocyanate intermediates, which are generated from acyl chlorides and KSeCN, resulting in the formation of 1,3,5-triazepineselone derivatives. This methodology offers great potential diversity and available starting materials [70]. This process is illustrated in Scheme 14.

**Scheme 15:**
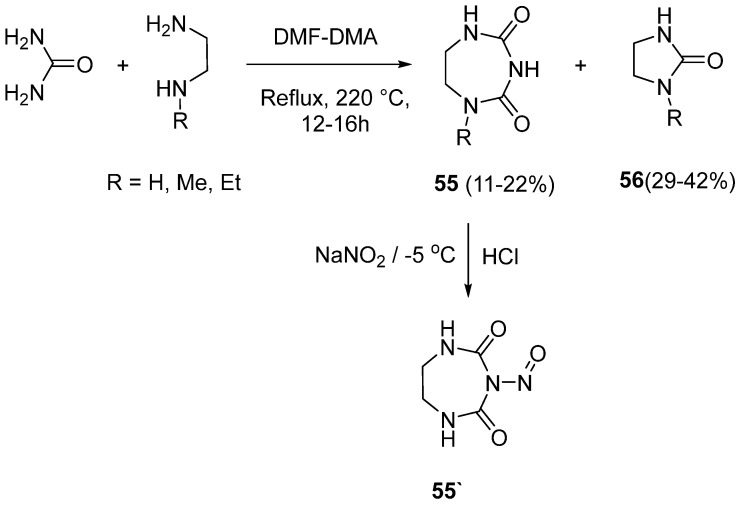
Synthesis of 1,3,5-triazepane-2,4-dione and imidazolidin-2-one and 3-nitroso-1,3,5-triazepane-2,4-dione derivatives.

The 1,3,5-triazepane-2,4-dione (**55**) and imidazolidin-2-one (**56**) derivatives (R = H, Me, and Et) were obtained by condensing ethane-1,2-diamine derivatives with urea in dimethylformamide-dimethyl acetal (DMF-DMA) under reflux conditions for a long time. Also, compound 3-nitroso-1,3,5-triazepane-2,4-dione (**55′**) was synthesized by coupling compound (**55**) with sodium nitrite and HCl under controlled stirring and cooling (−5 °C). These products, dihydro-3-nitroso-1*H*-1,3,5-triazepine-2,4(3*H*,5*H*)-dione (**55′**) and (**55**), have antitumor effects against AH-13 and L-1210 cells [71,72], as shown in Scheme 15.

**Scheme 16:**
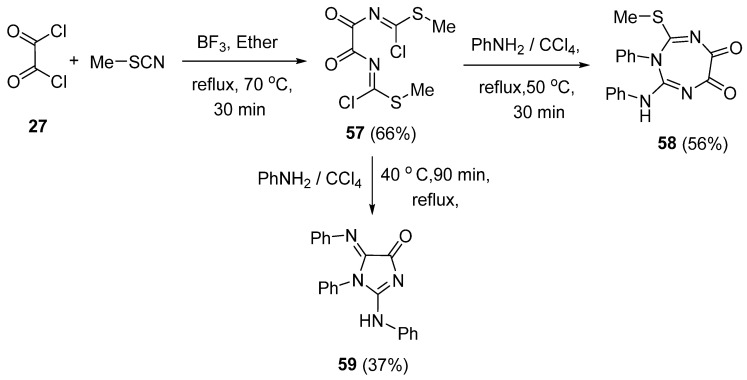
Synthesis of 2-(methylthio)-3-phenyl-4-(phenylamino)-3*H*-1,3,5-triazepine-6,7-dione derivatives.

The reaction of oxalyl dichloride (**27**) with thiocyanatomethane (MeSCN) at 1:2 molar ratio gave dimethyl oxalyl (*Z*,*Z*)-bis(carbonochloridoimidothioate) (**57**). The reaction of compound (**57**) with excess aniline gave the 2-(methylthio)-3-phenyl-4-(phenylamino)-3*H*-1,3,5-triazepine-6,7-dione (**58**) and 1-phenyl-2-(phenylamino)-5-(phenylimino)-1,5-dihydro-4*H*-imidazol-4-one (**59**) derivatives, respectively [73], as shown in Scheme 16.

**Scheme 17:**
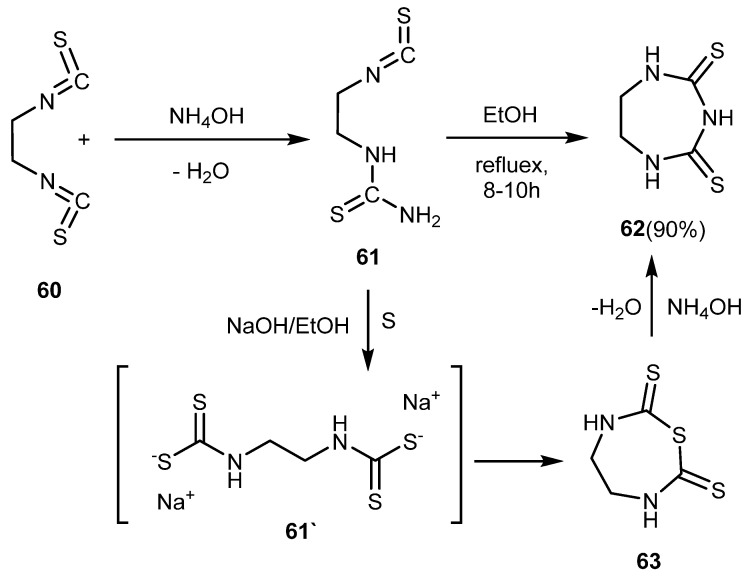
Synthesis of 1,3,5-triazepane-2,4-dithione/hexahydro-1,3,6-thiadiazepine-2,7-dithione derivatives.

The compound 1,3,5-triazepane-2,4-dithione (**62**) can be synthesized through two methods. In the first method, ethylenebisisotfhiocyanate (**60**) is reacted with ammonia solution (ammonium hydroxide) to yield 1-(2-isothiocyanatoethyl) thiourea (**61**), which is then cyclized with ethanol to produce 1,3,5-triazepane-2,4-dithione (**62**). In the second method, 1-(2-isothiocyanatoethyl) thiourea (**61**) reacts with the sulfur element in solution ethanol with sodium hydroxide to form an intermediate disodium ethylene-bisdithiocarbamate (**61′**), which is then cyclized into hexahydro-1,3,6-thiadiazepine-2,7-dithione (**63**). Also, 1,3,5-triazepane-2,4-dithione (**62**) is obtained by using an ammonia solution (NH_4_OH) with compound (**63**) [74]. These compounds have high antifungal activity [74], as demonstrated in Scheme 17.

**Scheme 18:**
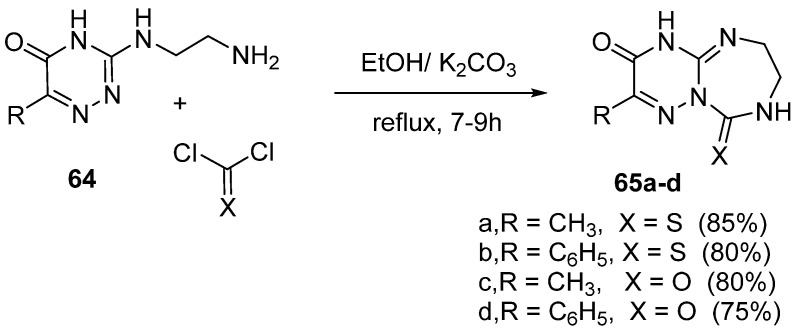
Synthesis of 1,2,4-triazino[3,2-*b*][1,3,5]triazepine-2,6-dione and 3-methyl-6-thioxo-6,7,8,9-tetrahydro-[1,2,4]triazino [3,2-*b*][1,3,5]triazepin-2(1*H*)-one derivatives.

The derivatives of the compounds 3-methyl-1,7,8,9-tetrahydro-[1,2,4]triazino[3,2-*b*] [1,3,5]triazepine-2,6-dione and 3-methyl-6-thioxo-6,7,8,9-tetrahydro-[1,2,4]triazino[3,2-*b*] [1,3,5]triazepin-2(1*H*)-one (**65a**–**d**) were prepared by treating phosgene or thiophosgene with 3-((2-aminoethyl) amino)-6-methyl-1,2,4-triazin-5(4*H*)-one resulting from using refluxing in ethanol cyclization with the formation of 1,3,5-triazepine (**65a**–**d**) derivatives [75], as demonstrated in Scheme 18.

### 2.5. From Pyridine N-Oxides and Imidazole Derivatives

**Scheme 19:**
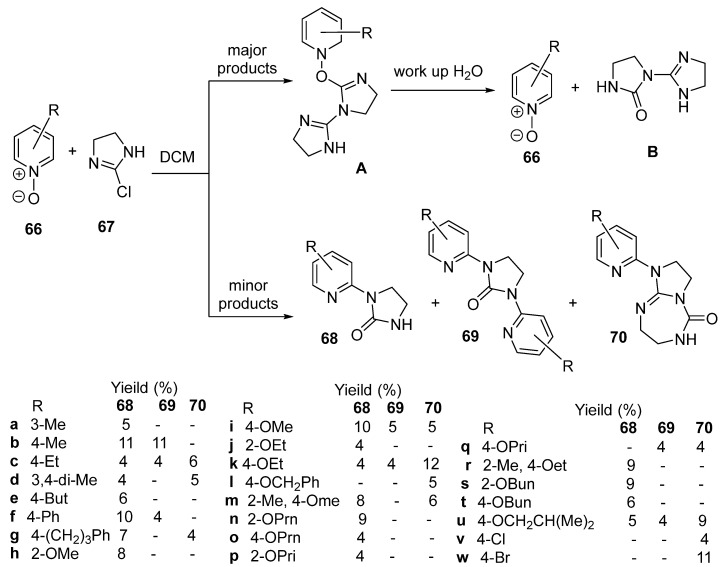
Synthesis of 1-(2-pyridyl)-2,3,7,8-tetrahydroimidazo[1,2-*b*][1,3,5]-triazepin-5(6*H*)-ones from the α-ureation and α-amination reactions of pyridine *N*-oxides with 2-chloro-imidazole.

Two types of chemical compounds were synthesized: 1-(2-pyridyl) imidazolidin-2-one and 1-(2-pyridyl)-2,3,7,8-tetrahydro-1*H*-imidazo[2,1-*b*][1,3,5]triazepin-5(6*H*)-one derivatives. These were obtained by subjecting substituted pyridine or quinoline *N*-oxides and 2-chloro-4,5-dihydroimidazole to ureation and amination reactions. The ureation reaction of pyridine and quinoline *N*-oxides was analyzed through quantum chemical calculations at the density functional theory level. The compounds (**68**–**70**) were tested for their in vitro cytotoxic activity against human tumor cell lines LCLC-103H, 5637, and A-427 [76]. In the process described earlier, where 2-aminopyridine *N*-oxide was combined with heteroarylation, the ureation process of alkyl-, alkoxy-, and halo-substituted pyridine *N*-oxides (**66**) with 2-chloro-4,5-dihydroimidazole (**67**) resulted in complex mixtures of products, including pyridinium salts of type (**A**). These salts reverted to the substrates (**66**) and *N*-(imidazolin-2-yl) imidazolidin-2-one (**B**) upon workup in an aqueous solution. In addition, we were able to isolate the desired α-ureation products, such as 1-monosubstituted imidazolidin-2-ones (**68**) and 1,3-disubstituted imidazolidin-2-ones (**69**), in 4–11% of the isolated yield using preparative thin-layer chromatography or column chromatography. Occasionally, from a reaction mixture, we also obtained 1-(2-pyridyl)-2,3,7,8-tetrahydroimidazo[1,2-*b*][1,3,5]-triazepin-5(6*H*)-ones (**70**) resulting from α-amination reaction of pyridine *N*-oxides with (**66**) [76]. The process is shown in Scheme 19.

**Scheme 20:**
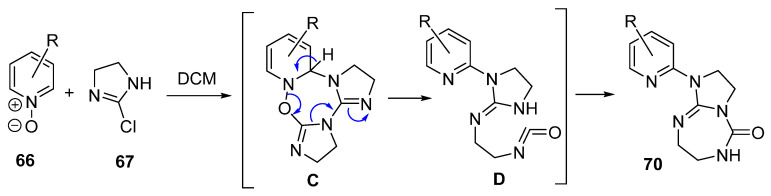
Proposed mechanisms for the α-amination reaction of pyridine *N*-oxides with 2-chloro-4,5-dihydroimidazole.

Proposed mechanisms [76] for the α-amination of derivative products (**70**) are depicted below. It is presumed that the initially formed unstable pyridinium salt (**A**) undergoes an α-amination reaction to give the dihydropyridine (**C**), which then undergoes a spontaneous 1,5-proton shift accompanied by re-aromatization of the dihydropyridine moiety to generate the isocyanate derivative (**D**). The intramolecular addition of the imidazolidine (NH) group to the heterocumulene then affords the final imidazo[1,2-*b*][1,3,5]-triazepinone (**70**) [76], as shown in Scheme 20.

**Scheme 21:**
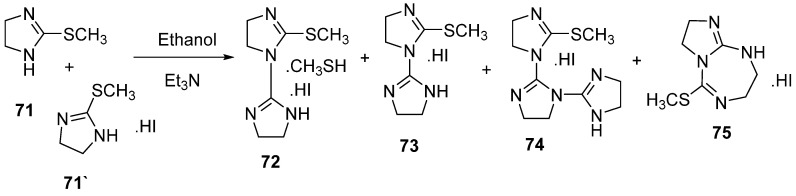
Synthesis of 5-(methylthio)-3*H*-imidazo[2,1-*b*][1,3,5]triazepine hydroiodide.

Treatment of 2-(methylthio)-4,5-dihydro-1*H*-imidazole (**71**) with HI gave 2-(methylthio)-4,5-dihydro-1*H*-imidazole hydroiodide (**71′**). The reaction of (**71**) and (**71′**) in ethanol with Et_3_N gave several products, including 1-(2-imidazolin-2-yl)-2-(methylthio)-2-imidazoline hydroiodide methanethiol (**72**), 2-(methylthio)-4,4′,5,5′-tetrahydro-1′H-1,2′-biimidazole hydroiodide (**73**), 2-(methylthio)-4,4′,4″,5,5′,5″-hexahydro-1″H-1,2′:1′,2″-terimidazole hydroiodide (**74**), and 5-(methylthio)-2,7,8,9-tetrahydro-3*H*-imidazo[2,1-*b*][1,3,5]triazepine hydroiodide (**75**) [77], as shown in Scheme 21.

**Scheme 22:**
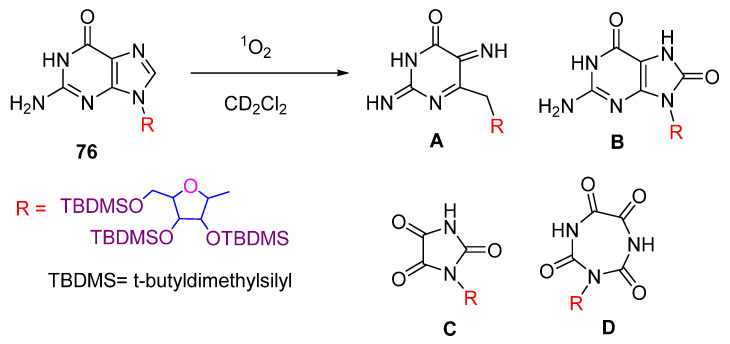
Synthesis of 1,3,5-triazepane-2,4,6,7-tetraone derivatives.

A guanosine derivative called 2-amino-9-(substituted-furan-2-yl)-1,9-dihydro-6*H*-purin-6-one (**76**) that dissolves in organic solvents was prepared [72]. The derivative was subjected to photosensitized oxidation in various solvents at different temperatures. The reactive oxidizing agent responsible for this reaction was singlet oxygen. However, no endoperoxide nor dioxetane intermediate was noticed by low-temperature NMR, even at −78 °C. Only one major product, 2,5-diimino-6-((substituted-ofuran-2-yl) methyl)-2,5-dihydropyrimidin-4(3*H*)-one (**A**), with an oxidized imidazole ring was detected at room temperature, which could be isolated by low-temperature column chromatography and was characterized by ^1^H and ^13^C and mass spectroscopy. Another major product was CO_2_. A small quantity of the corresponding 8-oxo-7,8-dihydroguanosine derivative, 2-amino-7,9-dihydro-1*H*-purine-6,8-dion**e** (**B**), was detected during the initial stage of photo-oxidation, which was an intermediate in the formation of two extensive degradation products, imidazolidine-2,4,5-trione (**C**) and 1,3,5-triazepane-2,4,6,7-tetraone (**D**) derivatives [78], as shown in Scheme 22.

### 2.6. From Active Methylene Compounds (Malononitrile Derivatives)

**Scheme 23:**
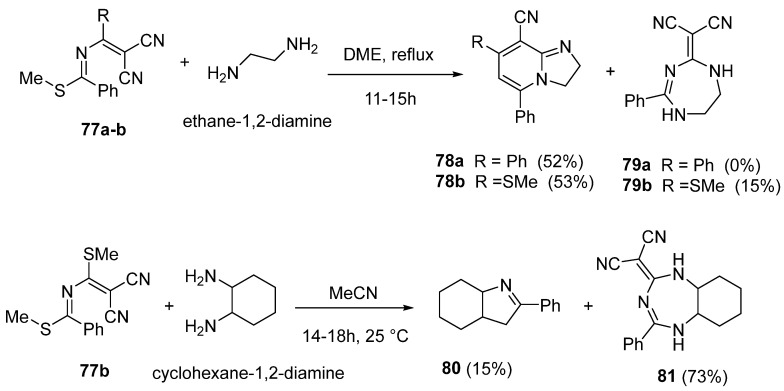
Synthesis of 1,3,5-triazepin-2-ylidene malononitrile and imidazopyrimidines derivatives.

Compounds (**77a**,**b**) were reacted with ethane-1,2-diamine in DMF to synthesize imidazo[1,2-*c*]pyrimidines (**78a**) and (**78b**) and triazepines (**79a**) and (**79b**), respectively. Additionally, the reaction of (**77b**) with cyclohexane-1,2-diamine in acetonitrile produced 2-phenyl-3a,4,5,6,7,7a-hexahydro-3*H*-indole (**80**) and 2-(4-phenyl-1,5,5a,6,7,8,9,9a-octahydro-2*H*-benzo[*f*][1,3,5] triazepin-2-ylidene) malononitrile (**81**) [79], as shown in Scheme 23.

**Scheme 24:**
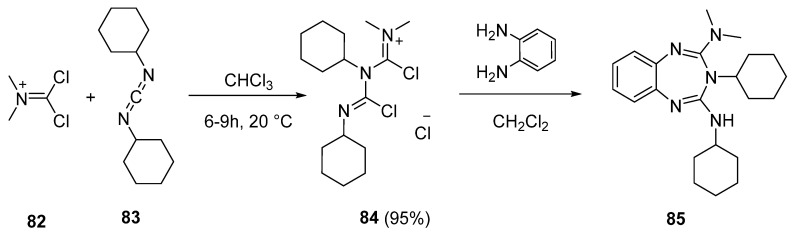
Synthesis of *N*^2^,3-dicyclohexyl-*N*^4^,*N*^4^-dimethyl-3*H*-benzo[*f*][1,3,5]triazepine-2,4-diamine derivatives.

In this chemical reaction, *N*-(dichloromethylene)-*N*-methylmethan-aminium (**82**) was mixed with dicyclohexyl-methane diimine (**83**) in chloroform. This resulted in the formation of *N*-(chloro (chloro(cyclohexylimino) methyl) (cyclohexyl) amino) methylene)-*N*-methylmethan-aminium chloride (**84**). Later, the same compound (**84**) was condensed with benzene-1,2-diamine, which led to the production of *N*^2^,3-dicyclohexyl-*N*^4^,*N*^4^-dimethyl-3*H*-benzo[*f*][1,3,5]triazepine-2,4-diamine (**85**) [80]. This chemical process is illustrated in Scheme 24.

### 2.7. From Isothiocyanates Derivatives

**Scheme 25:**
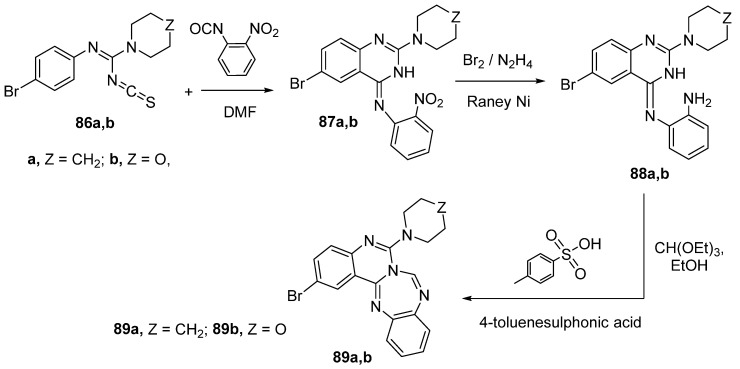
Synthesis of 12-bromo-6-(piperidine)benzo[6,7][1,3,5]triazepino-quinazoline and 4-(2-bromobenzo[6,7][1,3,5]triazepinoquinazoline) morpholine derivatives.

Upon heating, amidinoyl isothiocyanates (**86a**,**b**) react with 2-nitrophenyl isothiocyanate and undergo cyclization to form 4-(2′-nitroanilino) quinazolines (**87a**,**b**). The resulting compounds are then reduced to their 2′-amino derivatives (**88a**,**b**). Similarly, when *N*-(4-bromophenyl) piperidine-1-carbimidoyl isothiocyanate (**86a**) or *N*-(4-bromophenyl) morpholine-4-carbimidoyl isothiocyanate (**86b**) are heated with 2-nitrophenyl isothiocyanate in dimethylformamide, they form 2-piperidino-6-bromo-4-(2′-nitroanilino) quinazoline (**87a**) and 2-morpholino-6-bromo-4-(2′-nitroanilino) quinazoline (**87b**), respectively. The corresponding 2′-amino derivatives (**88a**,**b**) can be obtained by selectively reducing the nitro group of (**87a**,**b**) while conserving bromine using hydrazine and catalytic amounts of Raney nickel, following the procedure described in [81]. Finally, heating the 2′-amino derivatives (**88a**,**b**) with ethyl orthoformate and catalytic amounts of 4-toluenesulphonic acid leads to the formation of 12-bromo-6-(piperidin-1-yl)benzo[6,7][1,3,5]triazepino[3,2-*c*]quinazoline (**89a**) and 4-(2-bromobenzo[6,7][1,3,5]triazepino[3,2-*c*]quinazolin-6-yl)morpholine (**89b**), respectively [81], as shown in Scheme 25.

### 2.8. From Pyrazole Derivatives

**Scheme 26:**
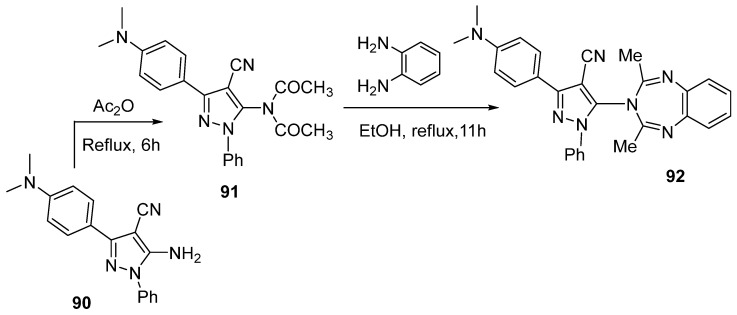
Synthesis of 5-(2,4-dimethyl-3*H*-benzo[*f*][1,3,5]triazepin-3-yl)-3-(4-(dimethyl-amino) phenyl)-1-phenyl-1*H*-pyrazole-4-carbonitrile.

Using microwave techniques to prepare heterocyclic compounds has been previously reported. Microwave irradiation is considered one of the green chemistry techniques due to improved yield, a safer environment, and reduced reaction times. Thus, when 5-amino-3-(4-(dimethyl-amino) phenyl)-1-phenyl-1*H*-pyrazole-4-carbonitrile (**90**) reacts with acetic anhydride, the *N*-acetyl-*N*-(4-cyano-3-(4-(dimethyl-amino)phenyl)-1-phenyl-1*H*-pyrazol-5-yl) acetamide (**91**) was obtained. The compound (**91**) was confirmed by its treatment with *o*-phenylenediamine to give 5-(2,4-dimethyl-3*H*-benzo[*f*][1,3,5]triazepin-3-yl)-3-(4-(dimethyl-amino) phenyl)-1-phenyl-1*H*-pyrazole-4-carbonitrile (**92**) [82]. Several pyrazoles (**91** and **92**) have demonstrated both antibacterial and anticancer properties [82], as shown in Scheme 26.

**Scheme 27:**
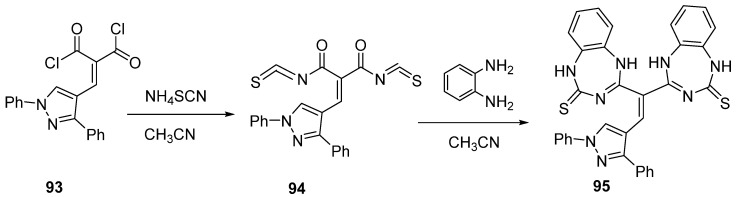
Synthesis of 4,4′-(2-(1,3-diphenyl-1*H*-pyrazole) ethene-1,1-diyl) bis (1,5-dihydro-2*H*-benzo[*f*][1,3,5]triazepine-2-thione).

Extensive research has been conducted on the pharmacological uses of pyrazole-based compounds which has led to the synthesis of a highly functionalized pyrazolylmalonyl diisothiocyanate derivative (**94**). The derivative was created by treating 2-(1,3-diphenyl-1*H*-pyrazol-4-yl) methylene) malonyl dichloride (**93**) with ammonium thiocyanate in acetonitrile at room temperature. This diisothiocyanate derivative (**94**) was then reacted with a double molar ratio of various nitrogen nucleophiles such as 4-acetylaniline, 2-aminobenzoic acid, 6-aminothiouracil, 2-aminothiadiazole derivative, hydrazine, phenylhydrazine, thiophene-2-carbohydrazide, 2-hydroxybenzohydrazide, 2-cyanoacetohydrazide, thiourea, thiosemicarbazide, 2-aminoaniline, and thiocarbohydrazide. This reaction aimed to construct valuable heterocyclic systems, such as the compound 4,4′-(2-(1,3-diphenyl-1*H*-pyrazol-4-yl) ethene-1,1-diyl) bis (1,5-dihydro-2*H*-benzo[*f*][1,3,5]triazepine-2-thione) (**95**). This compound was synthesized from 2-((1,3-diphenyl-1*H*-pyrazol-4-yl) methylene) malonyl diisothiocyanate (**94**) [83] with benzene-1,2-diamine. These compounds were screened for their insecticidal activity against healthy late third instar larvae *P. interpunctella* and *Nilaparvata lugens*. The results showed that benzo[*f*][1,3,5]triazepine-2-thione derivatives exhibited the highest insecticidal potency, which was supported by DFT stimulation and their molecular docking [83], as shown in Scheme 27.

### 2.9. From Thiazole Derivatives

**Scheme 28:**
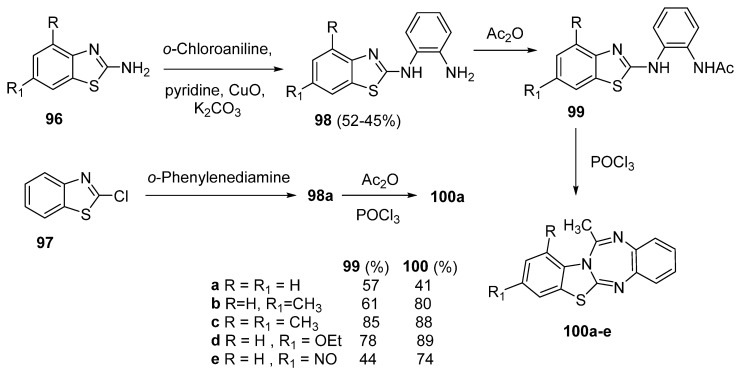
Synthesis of the 12-methylbenzo[*f*]benzo[4,5]thiazolo[2,3-*b*][1,3,5]triazepine.

A series of 3-substituted[1,2,4]triazepino[3,4-*b*]benzothiazolone (**100a**–**e**) compounds were synthesized through several steps. The synthesis process involved the reaction of benzo[*d*]thiazol-2-amine (**96**) or 2-chlorobenzo[*d*]thiazole with *o*-chloroaniline or *o*-phenylenediamine. Pyridine, potassium carbonate, and cupric oxide were added to the reaction mixture under the conditions of the Ullmann reaction to give substituted-*N*-(benzo[*d*]thiazol-2-yl) benzene-1,2-diamine (**98**). These *N*-phenylaminobenzothiazoles (**98**) were acetylated to give 2-(*o*-acylaminophenyl-amino) benzothiazoles (**99**), which underwent smooth cyclization in the presence of phosphorus oxychloride to produce the substituted-12-methylbenzo[*f*]benzo[4,5]thiazolo[2,3-*b*][1,3,5]triazepine (**100a**–**e**) [78]. An alternate approach involved reacting 2-chlorobenzothiazole (**97**) with *o*-phenylenediamine to give (**98a**). Several other derivatives were synthesized from 2-amino-6-methyl-, 2-amino-4,6-dimethyl-, 2-aminod-nitro-, and 2-amino-6ethoxy-benzothiazoles [84]. These compounds have the potential biological activity of simple triazepines [84], as shown in Scheme 28.

### 2.10. From Oxadiazole Derivatives

**Scheme 29:**
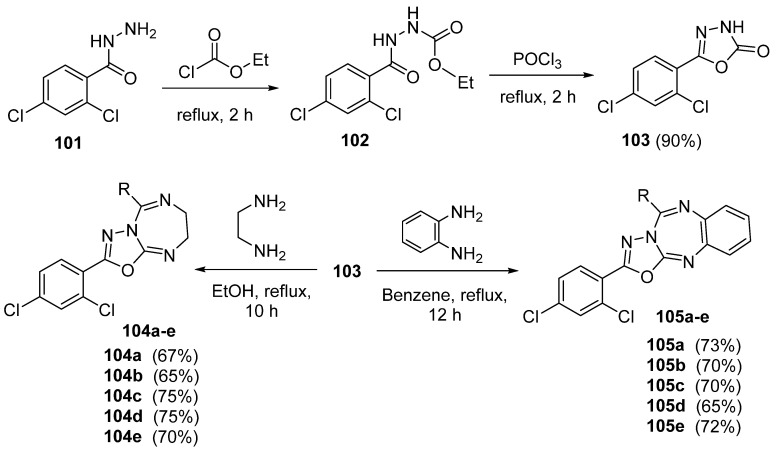
Synthesis of 2-(2,4-dichloro-phenyl)-[1,3,4]oxadiazolo[2,3-*b*][1,3,5]triazepine derivatives.

Two fused heterocyclic compounds (**104** and **105**), respectively [85], were synthesized through the cyclo-condensation of oxadiazoles (**103**) with ethylenediamine and *o*-phenylenediamine. The process involved the preparation of 2,4-dichlorobenzohydrazide (**101**), followed by the cyclo-condensation of ethyl carbonochloridate, which yielded ethyl 2-(2,4-dichlorobenzoyl) hydrazine-1-carboxylate (**102**). The latter was then phosphorylated using POCl_3_ to form 5-(2,4-dichlorophenyl)-1,3,4-oxadiazol-2(3*H*)-one (**103**). The essential compound (**103**) underwent cyclo-condensation with ethylenediamine or *o*-phenylenediamine, producing 2-(2,4-dichloro-phenyl)-7,8-dihydro-[1,3,4]oxadiazolo[2,3-*b*][1,3,5]triazepine (**104**) and 2-(2,4-dichlorophenyl) benzo[*f*][1,3,4]oxadiazolo[2,3-*b*][1,3,5]triazepine (**105**) derivatives [85], respectively, as shown in Scheme 29.

### 2.11. From Oxadiazepine Derivatives

**Scheme 30:**
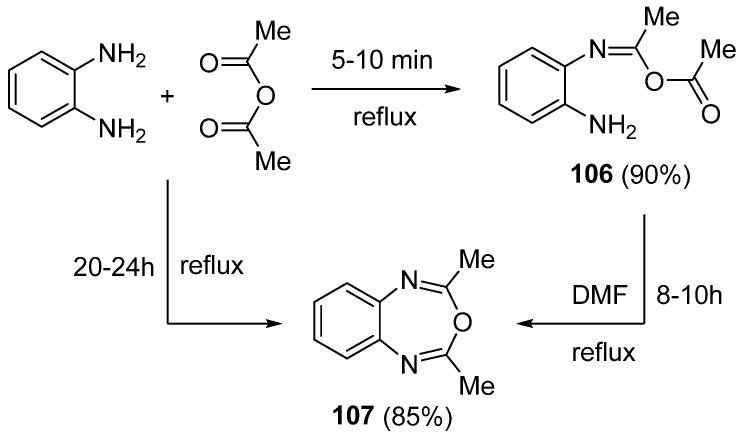
Synthesis of 2,4-dimethylbenzo[*d*][1,3,6]oxadiazepine derivatives.

After a few minutes of condensation of *o*-phenylenediamine with acetic anhydride, acetic-*N*-(2-aminophenyl) acetimidic anhydride (**106**) was formed in 90% yield. Further, the compound (**106**) was cyclized with dimethyl-formamide (DMF) under reflux for a long time, which resulted in the formation of 2,4-dimethylbenzo[*d*][1,3,6]oxadiazepine (**107**) in 85% yield. The same compound (**107**) was obtained through another method where a solution of *o*-phenylene-diamine and acetic anhydride was refluxed after condensation [86], as shown in Scheme 30.

**Scheme 31:**
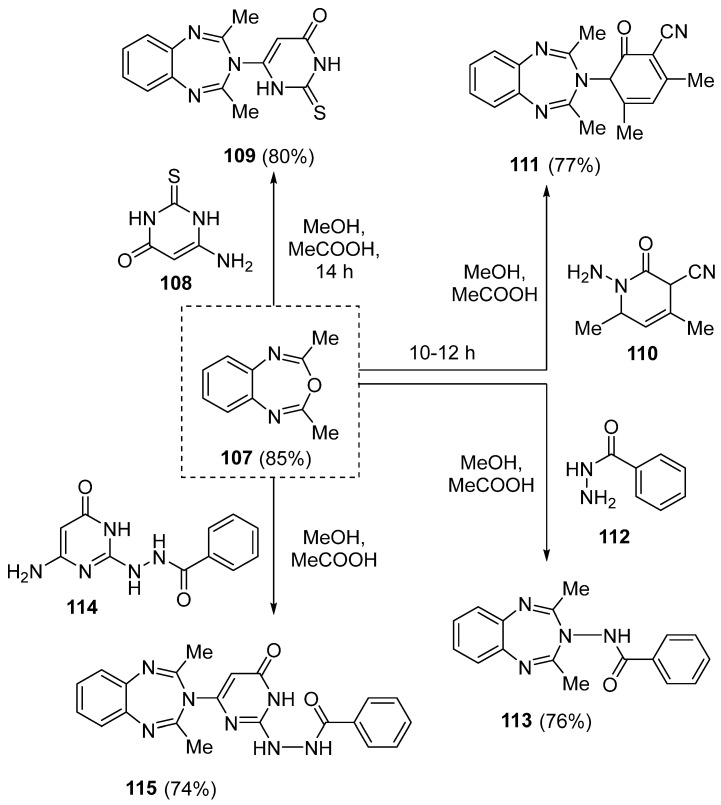
Preparation of benzo[*f*][1,3,5]triazepine derivatives, 74–80% yields.

A group of substituted 2,4-dimethyl-benzo[*f*][1,3,5]triazepine compounds were synthesized from 2,4-dimethyl benzo[*d*][1,3,6]oxadiazepine. These compounds were tested for their antitumor activities, and some of them showed promising results. The reactivity of compound (**107**) to amino group derivatives was also studied. The opening of the oxadiazepine ring followed by the elimination of (H_2_O) produced the triazepine ring. Treatment of compound (**107**) with numerous compounds in absolute methanol and a few drops of glacial acetic acid as the catalytic resulted in many new products including the following: 6-(2,4-dimethyl-benzo[*f*][1,3,5]triazepine)-2-thioxo-pyrimidinone (**109**) in 80% yield, 1-(2,4-dimethyl-benzo[*f*][1,3,5]triazepine)-4,6-dimethyl-2-oxo-pyridine-3-carbonitrile (**111**) in 77% yield, *N*-(2,4-dimethyl-benzo[*f*][1,3,5]triazepine)benzamide (**113**) in 76% yield, and *N*′-(4-(2,4-dimethyl-3*H*-benzo[*f*][1,3,5]triazepine)-6-oxo-1,6-dihydropyrimidine)benzohydrazide (**115**) in 74% yield, respectively [86], as shown in Scheme 31.

**Scheme 32:**
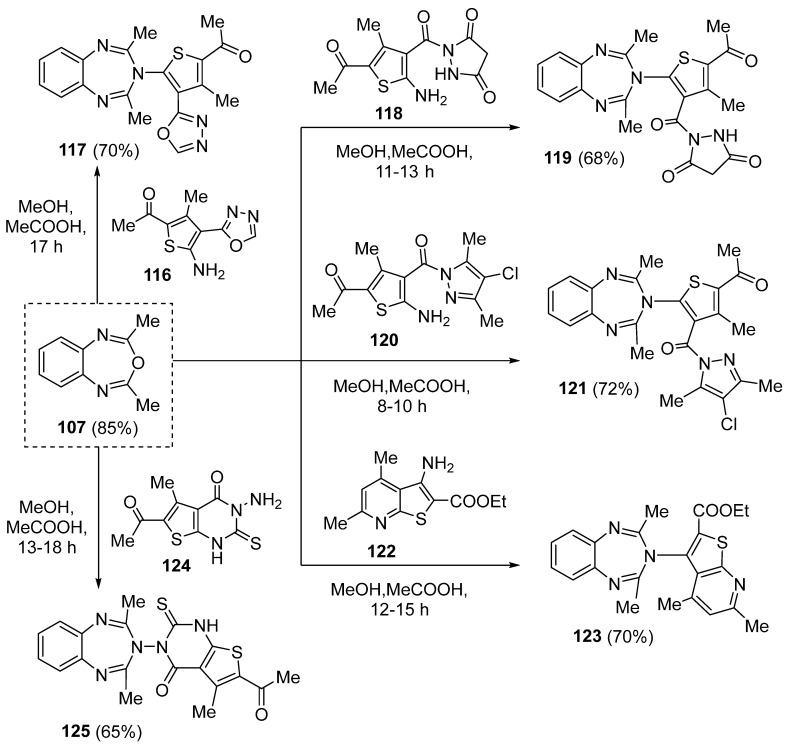
Preparation of benzo[*f*][1,3,5]triazepine from thiophene derivatives, 65–72% yields.

The following reactions were conducted to study the compound (**107**) and form heterocyclic compounds by reacting it with various thiophene derivatives. Treatment of (**107**) with 1-(5-amino-3-methyl-4-(1,3,4-oxadiazol-2-yl)thiophene) ethanone (**116**) in absolute methanol and a few drops of glacial acetic acid yielded 1-(5-(2,4-dimethyl-3*H*-benzo[*f*][1,3,5]triazepin-3-yl)-3-methyl-4-(1,3,4-oxadiazol-2-yl)thiophen-2-yl) ethan-1-one (**117**) with a 70% yield. Refluxing of compound (**107**) with 1-(5-acetyl-2-amino-4-methylthiophene-3-carbonyl) pyrazolidine-3,5-dione (**118**) in absolute methanol and a few drops of glacial acetic acid gave 1-(5-acetyl-2-(2,4-dimethyl-3*H*-benzo[*f*][1,3,5]triazepine)-4-methylthiophene-3-carbonyl) pyrazolidine-3,5-dione (**119**) with a 68% yield. The reaction of (**107**) with 1-(5-amino-4-(4-chloro-3,5-dimethyl-1*H*-pyrazole-1-carbonyl)-3-methylthiophene) ethan-1-one (**120**) in methanol and 2 mL of glacial acetic acid gave 1-(4-(4-chloro-3,5-dimethyl-1*H*-pyrazole-1-carbonyl)-5-(2,4-dimethyl-3*H*-benzo[*f*][1,3,5]triazepine) 3-methylthiophene) ethanone (**121**) with a 72% yield. Conducting a reaction of (**107**) with ethyl 3-amino-4,6-dimethylthieno[2,3-*b*]pyridine-2-carboxylate (**122**) in absolute methanol with glacial acetic acid as a catalyst yielded ethyl 3-(2,4-dimethyl-3*H*-benzo[*f*][1,3,5]triazepine)-4,6-dimethylthieno[2,3-*b*]pyridine-2-carboxylate (**123**) with a 70% yield. Reacting compound (**107**) with 6-acetyl-3-amino-5-methyl-2-thioxo-thieno[2,3-*d*]pyrimidinone (**124**) in absolute methanol and glacial acetic acid as a catalyst gave 6-acetyl-3-(2,4-dimethyl-3*H*-benzo[*f*][1,3,5]triazepine)-5-methyl-2-thioxo-2,3-dihydrothieno[2,3-*d*]pyrimidinone (**125**) with a 65% yield [86]. After being screened for cytotoxic activity in vitro, these compounds exhibited promising results [86], as shown in Scheme 32.

### 2.12. From Hydrazine Hydrate and Hydrazonoyl Chloride Derivatives

Several heterocyclic compounds have been synthesized from 6-(2,4-dimethyl-3*H*-benzo[*f*][1,3,5]triazepin-3-yl)-2-thioxo-2,3-dihydropyrimidin-4(1*H*)-one (**109**) and 6-(2,4-dimethyl-3*H*-benzo[*f*][1,3,5]triazepin-3-yl)-2-hydrazinyl-pyrimidin-4(3*H*)-one (**127**). The compounds were synthesized in moderate to excellent yields (61–95%) and included triazole, tetrazole, and spirocyclic (pyrimidine and thiadiazole) derivatives. The antimicrobial activity of these compounds against Gram-positive and Gram-negative bacteria and fungi was evaluated in vitro, and it was found that the benzo[*f*][1,3,5]triazepine derivatives showed potent antimicrobial activity against both Gram-positive and Gram-negative bacteria and fungi. The results were comparable to the positive controls levofloxacin and nystatin [87].

**Scheme 33:**
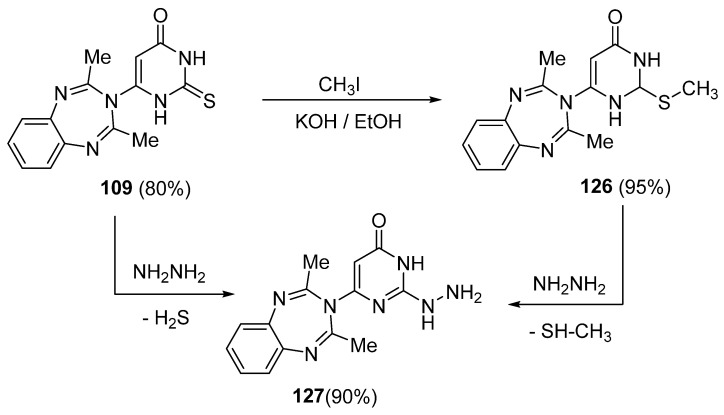
Synthesis of benzo[*f*][1,3,5]triazepine-2-(methylthio)-pyrimidinone and 2-hydrazinyl-pyrimidinone.

The alkylation of an ethanolic potassium hydroxide solution of 6-(2,4-dimethyl-3*H*-benzo[*f*][1,3,5]triazepin-3-yl)-2-thioxo-2,3-dihydropyrimidin-4(1*H*)-one (**109**) with methyl iodide resulted in the production of 6-(2,4-dimethyl-3*H*-benzo[*f*][1,3,5]triazepin-3-yl)-2-(methylthio) pyrimidin-4(3*H*)-one (**126**) in 95% yield. When treated with hydrazine hydrate, the compounds (**109**) or (**126**) provided 6-(2,4-dimethyl-3*H*-benzo[*f*][1,3,5]triazepin-3-yl)-2-hydrazinyl-pyrimidin-4(3*H*)-one (**127**), with the evolution of hydrogen sulfide or methyl mercaptan [87], as shown in Scheme 33. 

**Scheme 34:**
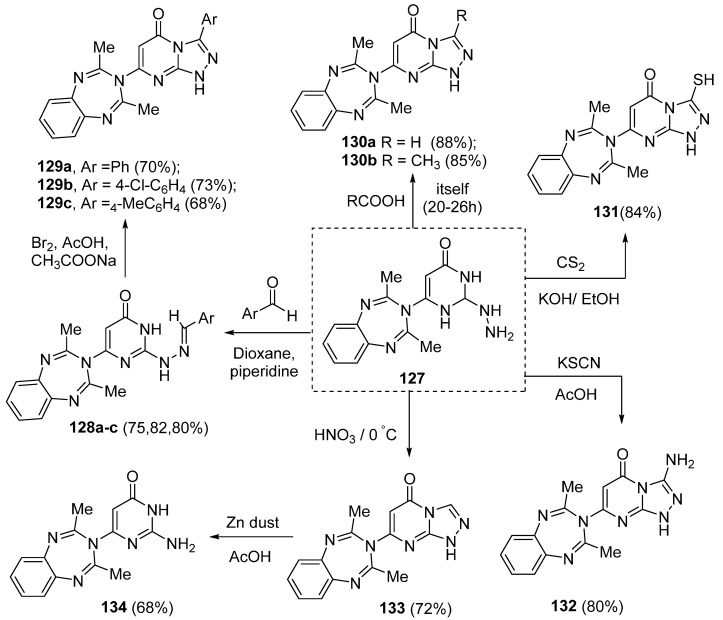
Preparation of benzotriazepine-1,2,4-triazolopyrimidinones and tetrazolopyrimidinones by using 2-hydrazinylpyrimidinones.

The compound hydrazinylpyrimidinone (**127**) is a versatile starting material that can be modified to synthesize various compounds including 1,2,4-triazolopyrimidinones, tetrazolo-pyrimidinones, and benzotriazepin-thiatetraazaspiro[4.5]decadienes. When hydrazinyl-pyrimidinone (**127**) is treated with the appropriate aldehyde in boiling glacial acetic acid, it yields 2-(2-(substituted-benzylidene) hydrazinyl)-6-(2,4-dimethyl-3*H*-benzo[*f*][1,3,5]triazepine) pyrimidin-4(3*H*)-ones (**128a**–**c**) in 75–82% yields. The aryl-hydrazones (**128a**–**c**) can then be cyclized to form 3-substituted-7-(2,4-dimethyl-3*H*-benzo[*f*][1,3,5]triazepine)-[1,2,4]triazolo[4,3-*a*]pyrimidin-5(1*H*)-one (**129a**–**c**) in 68–73% yields. This is achieved by treating the aryl-hydrazones with excess bromine in acetic acid in the presence of anhydrous sodium acetate under reflux. Compound (**127**) reacted with aliphatic acids such as formic or acetic acid, leading to the formation of 7-(2,4-dimethyl-3*H*-benzo[*f*][1,3,5]triazepine)-3-substituted-[1,2,4] triazolo[4,3-*a*]pyrimidinones, denoted as (**130a**,**b**) in yields of 88% and 85%, respectively [81]. Furthermore, when compound (**127**) was treated with carbon disulfide in an ethanolic potassium hydroxide solution, it produced 7-(2,4-dimethyl-benzo[*f*][1,3,5]triazepine)-3-mercapto-[1,2,4]triazolo[4,3-*a*]pyrimidinone, referred to as (**131**). The 2-hydrazino derivative (**127**) was treated with potassium thiocyanate in boiling acetic acid, resulting in the formation of 3-amino-7-(2,4-dimethyl-3*H*-benzo[*f*][1,3,5]triazepine)-[1,2,4]triazolo[4,3-*a*]pyrimidinone (**132**). When (**127**) was treated with nitrous acid at 0 °C, it led to the formation of 5-(2,4-dimethyl-3*H*-benzo[*f*][1,3,5]triazepine)tetrazolo[1,5-*a*]pyrimidinone (**133**). This latter compound (**133**) was then reduced to 2-amino-6-(2,4-dimethyl-3*H*-benzo[*f*][1,3,5]triazepine) pyrimidin-4(3*H*)-one (**134**) using zinc dust and acetic acid [87], as shown in Scheme 34.

**Scheme 35:**
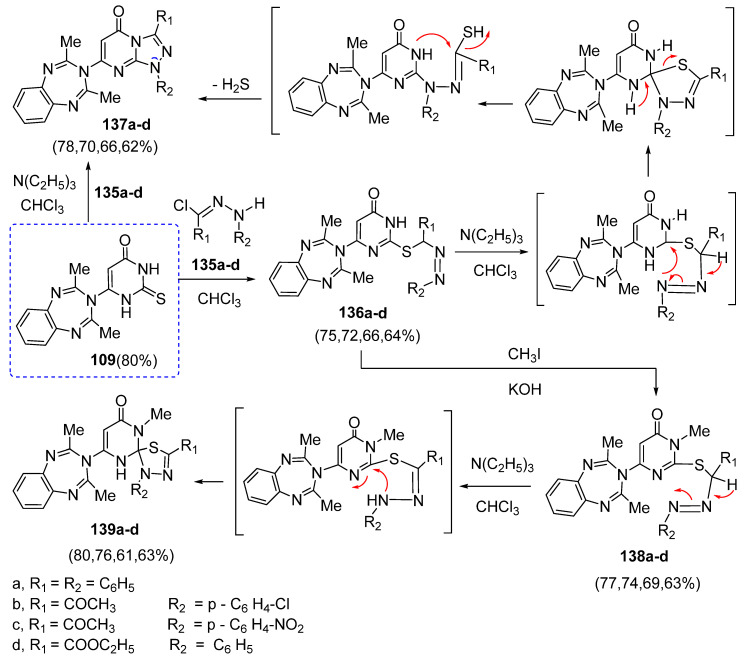
Preparation of 2,4-dimethyl-benzo[*f*][1,3,5]triazepine-4-thia-1,2,6,10-tetraazaspiro[4.5]deca-2,8-dienone derivatives.

The compound 6-(2,4-dimethyl-benzo[*f*][1,3,5]triazepine)-2-thioxo-2,3-dihydropyrimidin-one (**109**) was mixed with hydrazonoyl chloride derivatives (**135a**–**d**) [87] in dry chloroform and stirred under reflux for 5–8 h. This resulted in the production of 6-(2,4-dimethyl-benzo[*f*][1,3,5]triazepine)-2-(phenyldiazenylmethylenethio)pyrimidinones (**136a**–**d**) [81]. Substituted 7-(2,4-dimethyl-3*H*-benzo[*f*][1,3,5]triazepine)-[1,2,4]triazolo[4,3-*a*]pyrimidinones (**137a**–**d**) were prepared by stirring and refluxing compound (**109**) with the appropriate hydrazonoyl chlorides (**135a**–**d**) [81]. The reaction passed through intermediates (**136a**–**d**) in dry chloroform and triethylamine was a catalyst for a long time (12–20 h). Substituted 6-(2,4-dimethyl-benzo[*f*][1,3,5]triazepine)-3-methyl-2-(phenyldiazenylmethylenethio)pyrimidinones (**138a**–**d**) were prepared through the alkylation of (**136a**–**d**) with methyl iodide and ethanolic KOH. To obtain the cyclized product substituted 9-(2,4-dimethyl-benzo[*f*][1,3,5] triazepine)-6-methyl-4-thia-1,2,6,10-tetraazaspiro[4.5]deca-2,8-dien-7-ones (**139a**–**d**) [81], compounds (**138a**–**d**) were stirred under reflux with a few drops of triethylamine in dry chloroform for 8–12 h. The compounds were tested in vitro for antimicrobial activity and showed good results [87], as shown in Scheme 35. 

### 2.13. From Carbohydrate (Monosaccharides) Derivatives

**Scheme 36:**
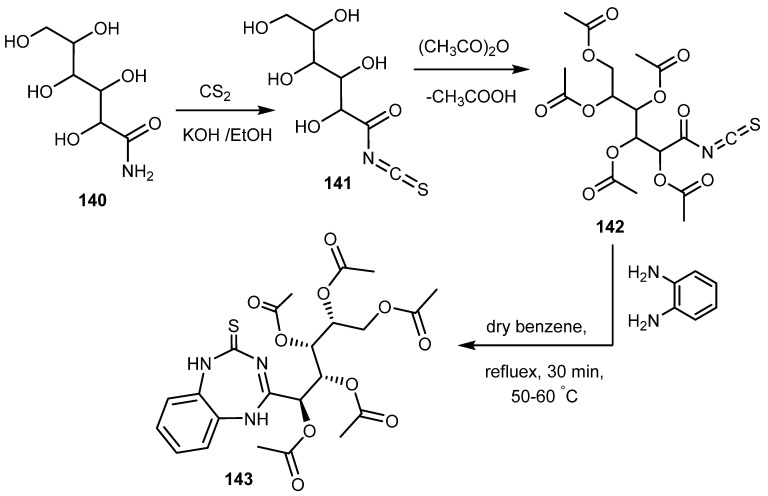
Preparation of 1,3,5-triazepine, benzo[*f*][1,3,5]triazepine derivatives.

A nucleoside derivative known as (1*S*,2*R*,3*R*,4*R*)-1-(4-thioxo-4,5-dihydro-1*H*-benzo[*f*][1,3,5]triazepin-2-yl) pentane-1,2,3,4,5-pentayl pentaacetate (**143**) [88] can be synthesized through various methods. One such method involves reacting 2,3,4,5,6-pentahydroxyhexanamide (**140**) with carbon disulfide in ethanolic potassium hydroxide to obtain 2,3,4,5,6-pentahydroxy-hexanoyl isothiocyanate (**141**). Acetic anhydride is then used to acylate compound (**141**) to form 6-isothiocyanato-6-oxohexane-1,2,3,4,5-pentayl pentaacetate (**142**). The resulting compound (**142**) is then condensed with *o*-phenylenediamine in dry benzene and heated to 50–60 °C for 30 min to yield the product (**143**) in good yield (84%). These compounds are known to have biological activity, including antibacterial, antiviral, and psychotropic activities [88], as shown in Scheme 36.

## 3. Summary of Biological Activity

This article provides a comprehensive review of more than 140 compounds of 1,3,5-triazepine and benzo[*f*][1,3,5]triazepine derivatives, including information on their preparation, reactions, and biological activity. This review is based on over 85 scientific references published in international journals. To make it easier to refer to, the biological activity of some compounds is presented in Table 1 and Table 2.

## 4. Conclusions

This review discusses the recent strategies in drug design that utilize seven-membered rings with three nitrogen atoms. By analyzing a few literature examples, it has been found that derivatives of seven-membered rings such as 1,3,5-triazepine and benzo[*f*][1,3,5] triazepine can act as bioisosteres to mono ortho-substituted biaryl systems. These rings offer new structures and vectors to explore in drug design. This review presents a comprehensive survey of the synthesis procedure, chemical reactions, and biological activities of 1,3,5-triazepine and benzo[*f*][1,3,5]triazepine derivatives. We have discussed several methods for the synthesis of these compounds, including 2-phenyl-benzo[*f*][1,3,5]triazepine; 2-methylene-benzo[*f*][1,3,5]triazepinol; substituted-phenyl-3*H*-benzo[*f*][1,3,5]triazepine-2-thiol; D-gluco-pentyl-benzotriazepine-2-thione, 5-phenyl-1*H*-benzo[*f*]imidazo[2,1-*b*][1,3,5]triazepine; benzo[*f*]imidazo[2,1-*b*][1,3,5]triazepin-5-one; 4-amino-2-(dimethylamino)-1,3,5-triazepinone; 3-sub-phenyl-2,4-diselenoxo-1,3,5-triazepane-6,7-dione; substituted-3-phenyl-2,4-dithioxo-1,3,5-triazepane-6,7-dione; 3-nitroso-1,3,5-triazepane-2,4-dione; 2-(methylthio)-3-phenyl-4-(phenylamino)-1,3,5-triazepine-6,7-dione; 1-(2-pyridyl)-tetrahydroimidazo[1,2-*b*][1,3,5]-triazepin-5(6*H*)-ones; 5-(methylthio)-3*H*-imidazo[2,1-*b*][1,3,5]triazepine hydroiodide; 1,3,5-triazepin-2-ylidene malononitrile; *N*^2^,3-dicyclohexyl-*N*^4^,*N*^4^-dimethyl-3*H*-benzo[*f*][1,3,5]triazepine-2,4-diamine; 12-bromo-6-(piperidine)benzo[6,7][1,3,5]triazepino-quinazoline; 4-(2-bromobenzo[6,7][1,3,5]triazepino-quinazoline) morpholine; 5-(2,4-dimethyl-3*H*-benzo[*f*][1,3,5]triazepine)-3-(4-(dimethyl-amino)phenyl)-phenyl-1*H*-pyrazole-4-carbonitrile; 12-methylbenzo[*f*]benzo[4,5]thiazolo[2,3-*b*][1,3,5]triazepine; 2-(2,4-dichloro-phenyl)-[1,3,4]oxadiazolo[2,3-*b*][1,3,5]triazepine; benzotriazepine-1,2,4-triazolopyrimidinones; and 2,4-dimethyl-benzo[*f*][1,3,5]triazepine-4-thia-1,2,6,10-tetraazaspiro[4.5]deca-2,8-dienone. We have referenced over (85) sources to provide a comprehensive overview. In the final part of this study, derivatives of quinazoline, pyrazole, thiazole, oxadiazole, triazolopyrimidine, and thiophene were highlighted. These derivatives were fused with a seven-membered ring, resulting in the formation of derivatives of quinazoline, pyrazole, thiazole, oxadiazole, and triazolopyrimidine which were linked with benzo[1,3,5]triazepine. These derivatives possess high biological activity, and this study suggests that exploring seven-membered ring analogues could be a promising approach for the development of new drugs.

## Figures and Tables

**Table 1:** Biological activity, bioactivity test, and organism/enzymes/substrates related to the biological assay for 1,3,5-triazepine and benzo[*f*][1,3,5]triazepine derivatives.

Compounds	Biological Activity	Bioactivity Test	Organism/Enzymes/Substrates	References
**18–19**	Antimicrobial activity	In vitro agar diffusion method	*E. coli*, *S. aureus*, *S. typhi*, *B. subtilis*, *A. aerogenes*, and *A. niger*	[61]
**109, 126** **–** **134, 136** **–** **139**	Antimicrobial activity	In vitro disc diffusion/micro dilution method	Bacteria: *Rhodo pseudomonas* fp., *Bacillus cereus*, *Micrococcus luteus*, *E. coli* (HD 701), and *Salmonella typhi;* fungi: *Alternaria alternata*, *Aspergillus flavus*, *Candida albicans*, and *Curvularia lunata*	[87]
**91** **–** **92**	Antibacterial activity	In vitro disc diffusion method	*E. coli*, *P. Aeuroginosa*, *S. Aureus*, and *B. Subtilis*	[82]
**62** **–** **63**	Antifungal activity	In vitro agar diffusion method	Many fungi such as Rhizopus	[74]
**94** **–** **95**	Insecticidal activity	In vitro	*P. interpunctella*, *Nilaparvata lugens*	[83]
**42** **–** **46**	Antihyperglycemic effect and reducing the formation of AGE	In vivo	Rats	[65,66,67]

**Table 2:** Biological activity, bioactivity test, and organism/enzymes/substrates related to the biological assay for 1,3,5-triazepine and benzo[*f*][1,3,5]triazepine derivatives.

Compounds	Biological Activity	Bioactivity Test	Organism/Enzymes/Substrates	References
**55′**, **55**	Antitumor activity	In vitro cytotoxicity assay	AH-13 cells and L-1210 cells	[71,72]
**91**–**92**	Anticancer activity	In vitro cytotoxicity assay	HCT-116 and MCF-7	[82]
**IC_50_** (μM) = **91**, **92**, respectively	**HCT-116** >100 μM, >100, respectively	**MCF-7.** 65.15 ± 3.7, 83.90 ± 4.6, respectively		
**51a**–**g**	Anticancer activity	In vitro cytotoxicity assay	MKN-45 gastric adenocarcinoma. Positive control = Paclitaxel drug (8.1 ± 0.02)	[69]
**IC_50_** (μM) = **51a**, **51b**, **51c**, **51d**, **51e**, **51f**, **51g**, respectively.	**MKN-45 gastric** 9.8 ± 0.01, 9.2 ± 0.02, 9.4 ± 0.01, 10 ± 0.01, 9.1 ± 0.01, 8.8 ± 0.01, 9.1 ± 0.01, respectively			
**68a–g**; **69a–g**; **70a–g**	Anticancer activity	In vitro cytotoxicity assay	LCLC-103H, 5637, and A-427 human cancer cell lines	[76]
**IC_50_** (μM) = **68a**, **68c**, **68g**, **68h**, **68j**, **68r**, **68s**, and **69i**, **69l**, and **70i**, **70q**, **70w**, respectively.	**LCLC-103H.** 137.04 ± 27.2, 101.2 ± 7.2, 52.87 ± 5.35, 101.2 ± 6.4, 96.7 ± 20.3, 100.5 ± 8.8, 93.4 ± 9.3, 74.1 ± 13.8, 58.97 ± 16.98, 99.3 ± 14.1, 96.8 ± 4.2, and 91.3 ± 5.2, respectively	**5637.** 129.44 ± 25.08, 95.9 ± 32.2, 87.6 ± 14.53, 126.8 ± 10.8, 23.9 ± 13.2, 130.3 ± 11.3, 124.3 ± 46.7, 25.9 ± 2.0, 82.9 ± 10.97, 86.5 ± 12.7, 57.6 ± 12.8, and 38.4 ± 4.7, respectively	**A-427.** 133.21 ± 24.14, 74.2 ± 7.3, 106.04 ± 1.6, 101.3 ± 6.7, 18.4 ± 7.7, 93.5 ± 27.7, 38.54 ± 9.8, 79.7 ± 13.3, 115.75 ± 13.72, 87.1 ± 20.1, 86.2 ± 22.1, and 38.4 ± 11.1, respectively	
**107**–**125**	Anticancer activity	Ehrlich in vitro assay	Cytotoxic evaluation Mortality (%)	[86]
**107**, **109**, **111**, **113**, **115**, **117**, **119**, **121**, **123**, **125**, respectively.	**Mortality (%)** 64.3 ± 0.034, 82.2 ± 0.001, 78.75 ± 0.022, 72.55 ± 0.024, 86.1 ± 0.002, 94.6 ± 0.004, 92.85 ± 0.008, 90.7 ± 0.045, 88.9 ± 0.003, and 96.5 ± 0.005, respectively			

## Data Availability

All of the data are included in this review article.

## Abbreviations

Be[*f*][1,3,5]Tri	Benzo[*f*][1,3,5]triazepine
TriPy	Triazolopyrimidines
TEA	Triethylamine
THF	Tetrahydrofuran
DDQ	2,3-dichloro-5,6-dicyano-1,2-benzoquinone
CDI	Carbonyldiimidazole
MCR	Multicomponent Reactions
DMSO	Dimethyl Sulfoxide
DMF	*N*,*N*-Dimethylformamide
TFA	Trifluoroacetic Acid
TBDMS	t-butyldimethylsilyl
